# Improved Cell-Potent and Selective Peptidomimetic Inhibitors of Protein N-Terminal Methyltransferase 1

**DOI:** 10.3390/molecules27041381

**Published:** 2022-02-18

**Authors:** Guangping Dong, Iredia D. Iyamu, Jonah Z. Vilseck, Dongxing Chen, Rong Huang

**Affiliations:** 1 Department of Medicinal Chemistry and Molecular Pharmacology, Purdue Institute for Drug Discovery, Purdue University Center for Cancer Research, Purdue University, West Lafayette, IN 47907, USA; dong216@purdue.edu (G.D.); iiyamu@purdue.edu (I.D.I.); chendoxi@hotmail.com (D.C.); 2 Department of Biochemistry and Molecular Biology, Center for Computational Biology and Bioinformatics, Indiana University School of Medicine, Indianapolis, IN 46202, USA; jvilseck@iu.edu

**Keywords:** protein N-terminal methyltransferase, peptidomimetic inhibitor, cell-permeable inhibitor, structure-based drug design

## Abstract

Protein N-terminal methyltransferase 1 (NTMT1) recognizes a unique N-terminal X-P-K/R motif (X represents any amino acid other than D/E) and transfers 1–3 methyl groups to the N-terminal region of its substrates. Guided by the co-crystal structures of NTMT1 in complex with the previously reported peptidomimetic inhibitor DC113, we designed and synthesized a series of new peptidomimetic inhibitors. Through a focused optimization of DC113, we discovered a new cell-potent peptidomimetic inhibitor GD562 (IC_50_ = 0.93 ± 0.04 µM). GD562 exhibited improved inhibition of the cellular N-terminal methylation levels of both the regulator of chromosome condensation 1 and the oncoprotein SET with an IC_50_ value of ~50 µM in human colorectal cancer HCT116 cells. Notably, the inhibitory activity of GD562 for the SET protein increased over 6-fold compared with the previously reported cell-potent inhibitor DC541. Furthermore, GD562 also exhibited over 100-fold selectivity for NTMT1 against several other methyltransferases. Thus, this study provides a valuable probe to investigate the biological functions of NTMT1.

## 1. Introduction

Protein α-N-terminal methyltransferases (NTMTs/NRMTs) catalyze the addition of 1–3 methyl group(s) to the protein α-N-termini from the cofactor *S*-adenosyl-*L*-methionine (SAM). Protein N-terminal methyltransferase 1/2 (NTMT1/2, METTL11A/B) are the two reported members of this family that recognize a unique N-terminal X-P-K/R motif (X represents any amino acid other than D/E) [1,2]. To date, the regulator of chromosome condensation 1 (RCC1), the tumor suppressor retinoblastoma 1 (RB1), oncoprotein SET, centromere protein A/B (CENP-A/B), damaged DNA-binding protein 2 (DDB2), poly(ADP-ribose) polymerase 3 (PARP3), Obg-like ATPase 1 (OLA1), and MORF-related gene on chromosome 15 (MRG15) have been reported as physiological substrates for NTMT1 [1,3,4,5,6,7,8]. Though protein α-N-terminal methylation is evolutionarily conserved across different species [8], the knowledge of its functions remains scant. Unlike aliphatic side-chain amines (pKa: 10.5), full methylation of the protein α-N-terminal amine (pKa: 6~8) would alter both the hydrophobicity and charge state [9]. The resulting positive charge at protein α-N-terminus strengthens the interactions of proteins to their respective binding partners, as exemplified in RCC1 and CENP-A [1,3,10]. Besides, the α-N-terminal methylation of DDB2 facilitates its nuclear localization to cyclobutane pyrimidine dimer (CPD) foci [4]. In addition, NTMT1 has been implicated in regulating mitosis, DNA damage repair, stem cell maintenance, and cervical cancer cell proliferation and migration [1,11,12,13]. Thus, cell-potent inhibitors would serve as valuable tools to study the functions and roles of NTMT1/2.

**Figure 1:**
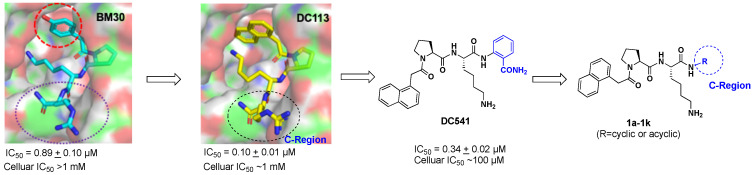
Optimization strategy of cell-potent peptidomimetic NTMT1 inhibitors.

Guided by the unique substrate-binding mode of NTMT1/2, we have reported several peptidomimetic inhibitors with modest cellular potency [14,15]. The first peptidomimetic inhibitor BM30 displayed an IC_50_ value of 0.89 ± 0.1 µM and over 100-fold selectivity for NTMT1/2 among a panel of 41 methyltransferases [14]. To improve the cellular uptake of peptidomimetic inhibitors [14], substituting the phenol group of BM30 with a naphthalene ring yielded DC113, with improved inhibition activity (IC_50_ = 0.1 ± 0.01 µM) and cell permeability (Figure 1) [15]. However, DC113 only decreased ~50% of the cellular me3-RCC1 level at 1 mM in HCT116 cells [15]. The co-crystal structure of NTMT1-DC113 (Figure 1) confirmed a similar binding mode of DC113 as the peptide substrate SPKRIA [15]. As the Pro2 and Lys3 moieties in the peptide substrates and peptidomimetic inhibitors are essential to the binding [14], the subsequent replacement of Arg4 of DC113 with benzamide provided DC541, enhancing cellular inhibition (IC_50_ of ~100 μM) on the me3-RCC1 level in HCT116 compared to DC113 [15]. Thus, we hypothesized that further introducing hydrophobic groups would promote cellular potency without perturbing the binding to NTMT1. Herein, we proposed a focused optimization on the C-region of DC113 or DC541 to enhance cellular potency (Figure 1).

## 2. Design

Compared to DC113, DC541 exhibited about 10-fold enhanced cellular inhibition with a ~3-fold decrease in its inhibitory activity on recombinant NTMT1 [15], suggesting that optimization of the C-region of the ligand scaffold is a feasible strategy to improve the hydrophobicity and enhance the cell potency of the compounds (Figure 1). As the C-region is exposed to solvent, we expect minimal interferences with the binding to NTMT1. In addition, incorporating non-amino acid functional groups has the potential to improve the compound’s proteolytic stability [15]. Therefore, we hypothesized that incorporating more hydrophobic groups at the C-region of DC541 would increase ligand hydrophobicity, permeability, and proteolytic stability without significantly affecting the binding to NTMT1. Thus, we introduced different R groups to increase the hydrophobicity considering its positive correlation to permeability [16], using cLogP as the parameter to track hydrophobicity in the design [17].

## 3. Synthesis

**Scheme 1:**
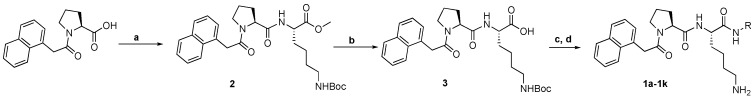
Synthetic route for peptidomimetic inhibitors **1a–k**.

All the peptidomimetic inhibitors were synthesized in the solution phase, as shown in Scheme 1 [15]. Compound **2** was synthesized through the standard amide coupling of HCl•Lys(Boc)-COMe with Naphthalene-Pro. Subsequent hydrolysis of **2** provided **3**. The final peptidomimetic inhibitors **1a**–**k** were prepared by the reactions of various amines with **3,** followed by the acidic treatment with 4 N HCl in 1,4-Dioxane.

Reagent and conditions: (a) HCl•Lys(Boc)-COMe, HBTU, HOBt, DIPEA, DMF, r.t.; (b) LiOH•H_2_O, MeOH/H_2_O, 0 °C to r.t.; (c) R-NH_2_, HBTU, HOBt, DIPEA, DMF, r.t.; (d) 4N HCl in dioxane, 0 °C to r.t.

## 4. Structure–Activity Relationship

Inhibitory activities of all synthesized compounds were determined with an established SAHH-coupled fluorescence assay [14,15]. To increase hydrophobicity, we first replaced the benzamide ring with a bromophenyl group to produce **1a** and **1b** with higher cLogP values than DC541 (Table 1). Both compounds showed ~2-fold increased inhibition compared to DC541, suggesting the bromide may form a halogen bond with NTMT1. Next, we varied the carbon length between the phenyl ring and the amide group to produce **1c**–**e**, displaying inhibitory activities with IC_50_ values ranging from 0.65 to 0.93 µM. The comparable inhibition of **1c**–**e** suggested a marginal effect of chain linker length on the inhibitory activity. To explore the importance of aromaticity at the C-region, we replaced the phenyl ring of **1c** with a cyclohexyl ring to produce **1g**, exhibiting a similar inhibition activity as **1c**. As the introduction of the bromide at the phenyl ring led to ~2-fold enhanced inhibition for **1a**,**1b** compared to DC541, we also added bromide to the phenyl ring of **1c** at the meta- and para- positions to generate **1g** and **1h**, respectively. However, **1g** and **1h** showed marginally improved potency (less than 2-fold) compared with **1c**. As N-methylation has been proven to improve the cell permeability of peptides [18], we introduced a methyl group to the C-terminal amide of **1d** to produce **1i,** which resulted in a 2-fold decreased inhibition. To explore the possible interaction with Tyr215 [15,19], we synthesized a biphenyl group at the C-terminal region to yield **1j** (IC_50_ = 2.5 ± 0.09 µM), resulting in a ~3-fold inhibition reduction compared to **1d**. Then, we switched the substitution position of the phenyl ring from the para to the meta position to produce **1k** (IC_50_ = 0.69 ± 0.08 µM), rescuing the inhibition with the comparable activity as the parent compound **1d**.

## 5. Computational Studies

**Figure 2:**
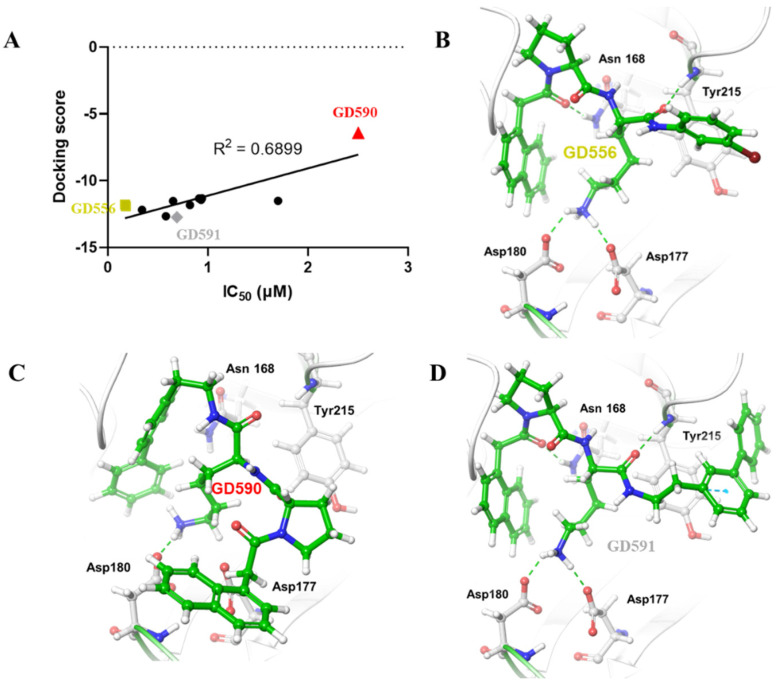
Docking studies of the peptidomimetic inhibitors through Glide. (**A**) Linear regression model of IC_50_ values and docking scores of the inhibitors. (**B**–**D**) Representative docking models of GD556, GD590, and GD591. The NTMT1 is shown in the color ribbon. The peptidomimetic inhibitors are shown in the green stick, and the interacting residues are shown in the gray stick. The H-bonds are shown in green–dotted lines.

To understand the contribution of different functional groups to the inhibition activities of the peptidomimetic inhibitors, we first performed a docking study using Glide software (Schrödinger) [19,20]. The IC_50_ values correlated well with the docking scores yielding an R^2^ of ~0.7 (Figure 2A). All peptidomimetic inhibitors except **1j** (GD590) docked with a similar binding mode as DC541 and DC113. For example, the ε-NH_2_ group of the Lys3 side chain in **1a** (GD556) formed electrostatic interactions with Asp177 and Asp180, while the backbone carbonyl group interacted with Tyr215 through a hydrogen bond (Figure 2B). In contrast, **1j** only interacted with Asp180 and lost the hydrogen bonding with Tyr215, providing a structural explanation for its eight-fold decrease in inhibition activity compared to DC541 (Figure 2C). In support of our hypothesis, the C-terminal biphenyl ring at the meta-position in compound **1k** (GD591) facilitated interactions with Asp 180, Asp 177, and Tyr215, with an additional π-π interaction between the fourth position phenyl ring and Tyr215 (Figure 2D).

**Figure 3:**
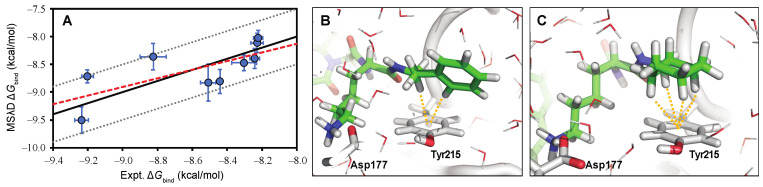
Computational studies through alchemical free energy calculations. (**A**) Correlation between MSλD computed and experimental binding affinities for DC541 and **1a**–**h**. The best fit line (red, dashed) matches well the ideal y = x line (black, solid). Trajectory snapshots highlight π stacking and CH—π interactions (orange, dashes for **1d** (**B**) and **1f** (**C**)).

Alchemical free energy calculations, which employ a flexible receptor and explicit solvation, were performed next with multisite λ-dynamics (MSλD) for a subset of compounds (**1a**–**h**) [21,22]. A good correlation between computed and experimental binding free energies (Δ*G*_bind_) was again observed with a Pearson R of 0.71 (Figure 3, Table S1). Experimental Δ*G*_bind_ were estimated from the IC_50_ values in Table 1 [23]. Compounds **1a**,**1b** were modeled both with and without σ-hole lone pair particles to investigate the importance of halogen bonding for these compounds. However, no significant difference was observed in computed Δ*G*_bind_, suggesting halogen bonding does not play a dominant role in binding. In contrast, all C-region phenyl rings interacted strongly with Tyr215 and formed T-shaped and offset-parallel π-π stacking conformations. Thus, the benefit of the bromine atoms in **1a**,**1b** likely stems from its electron-withdrawing and increased dispersive character, which are known to strengthen π-π stacking interactions [24]. In molecules **1c**–**e**, as the carbon linker was elongated, ideal π-π stacking interactions and geometries were disrupted, slightly weakening ligand complexation. For example, the optimal center of mass (COM) distances between stacked benzene rings are often ~5 Å apart [25]. Figure S1 shows a positive trend between experimental Δ*G*_bind_ and increasing COM distances between substituent rings and Tyr215, measured from the MSλD trajectories. Surprisingly, as the carbon linker was extended, the consistent orientation of the methylene hydrogens pointed towards the π surface of Tyr215 (Figure 3). Though weaker than conventional hydrogen bonds, CH—π bonds have been shown to present weak attractive forces [26]. The equivalency in Δ*G*_bind_ between **1c, 1d**, and **1f** suggests that these CH–π interactions may help sufficiently counteract the loss of π-stacking with Tyr215 through favorable dispersive interactions and hydrophobic packing.

## 6. Cytotoxicity

**Figure 4:**
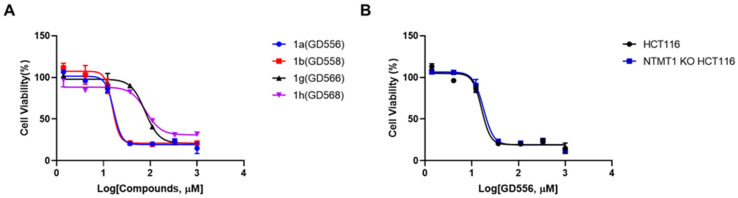
Cytotoxicity studies of the peptidomimetic inhibitors via alamarBlue assay (*n* = 3). (**A**) The effects of **1a**, **1b**, **1g**, and **1h** on growth in normal HCT116 cells. (**B**) The effects of **1a** (GD556) on growth in both normal and NTMT1 knockout HCT116 cells.

We evaluated the cytotoxicity of those peptidomimetic inhibitors (IC_50_ < 2 μM) in HCT116 cells through alamaBlue assay, as HCT116 cells are known for their high expression of NTMT1 [15]. Compared with DC541, all bromide compounds (**1a**, **1b**, **1g,** and **1h**) and **1k** displayed cytotoxicity, with GI_50_ values ranging from 48 μM to 135 μM (Table 2, Figure 4A). Among them, **1a**, **1b**, and **1k** exhibited GI_50_ values of <70 μM, while **1g** and **1h** showed moderate growth inhibition against HCT116 with GI_50_ values of ~130 μM. All other compounds showed negligible inhibition of cell growth even at 300 μM. As NTMT1 knockout did not exhibit any significant inhibition on HCT116 cell growth [27], we reasoned that the inhibition of NTMT1 would not affect the HCT116 cell growth. Thus, we hypothesized that the growth inhibition effects of these five peptidomimetic inhibitors may be caused by off-target effects. To test our hypothesis, we selected **1a** to evaluate its impact on both wild-type and NTMT1 knockout HCT116 cells (Figure 4B) [27]. Compound **1a** exhibited a comparable GI_50_ value on HCT116 cell growth, regardless of the presence of NTMT1, suggesting the growth inhibition effect resulted from off-target effects.

## 7. Inhibition on Cellular N-Terminal Methylation

**Figure 5:**
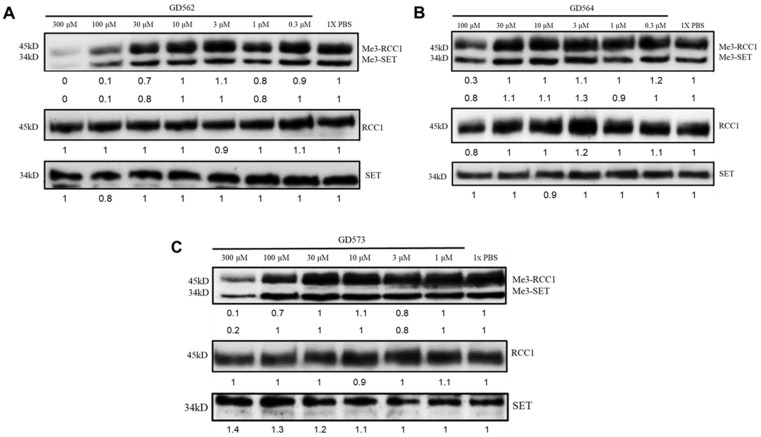
Inhibition of cellular N-terminal methylation by peptidomimetic inhibitors in HCT116 cells. Representative Western blot results of the effects of GD562 (**A**), GD564 (**B**), and GD573 (**C**) on the cellular methylation level. Image quantification was performed using ImageJ software (NIH). All bands were compared to the respective untreated control, which was set at 1.0. All experiments were performed in duplicate (*n* = 2).

Next, we investigated the effects of all peptidomimetic inhibitors with GI_50_ > 100 µM and IC_50_ < 1 µM on the cellular α-N-terminal methylation level in HCT116 cells (Table 2) [15]. Then, we examined the top three cell-potent peptidomimetic inhibitors **1d**–**f** on the dose-response of inhibition of cellular N-terminal methylation levels. Compared to compound DC541 [15], **1d**–**f** displayed comparable or improved cellular inhibition activities on both me3-RCC1 and me3-SET (Figure 5A–C). Among them, **1d** (GD562) exhibited improved cellular inhibition on me3-RCC1 with an IC_50_ of ~50 µM (Figure 5A). Furthermore, GD562 also inhibited me3-SET with a similar cellular IC_50_ value of ~50 µM, while DC541 did not show any inhibition of me3-SET level even at 300 µM in HCT116 cells [15]. As the biochemical IC_50_ value of GD562 was about three-fold higher than DC541, we proposed that the improved cellular inhibition of GD562 may be attributed to its increased permeability with higher cLogP than DC541. 

## 8. Permeability

To understand the discrepancy between biochemical IC_50_ and cellular IC_50_ values, we evaluated the ability of these inhibitors to cross the cell membrane in the Parallel Artificial Membrane Permeability Assay (PAMPA) [28,29,30]. The permeability of the control compound verapamil was determined as 8.6 ± 0.98 × 10^−6^ cm/s, comparable to the literature value (8.8 ± 0.53 × 10^−6^ cm/s) [30]. As shown in Table 2, the majority of synthesized peptidomimetics showed 10- to 50-fold lower permeability compared to verapamil. The phenyl analogs **1c**–**e** with variable carbon length showed the lowest permeability values ranging from 0.17 × 10^−6^ cm/s to 0.26 × 10^−6^ cm/s, which was about two-fold less than the cyclohexyl analog **1f** with a permeability value of 0.47 × 10^−6^ cm/s. This suggests that acyclic rings have better cell permeability than aromatic rings. Methylation of the C-terminal amide of **1d** to **1i** resulted in a higher cLogP, which was also evident in a roughly two-fold improvement in cell permeability. As expected, the biphenyl analogs **1j** and **1k** had the higher cell permeability values of 0.9 × 10^−6^ cm/s and 1.3 × 10^−6^ cm/s due to the improved lipophilicity of the additional phenyl group. This trend of the permeability values from the PAMPA assay is proportional to that of the cLogP values, with **1j** and **1k** displaying the highest cLogP values of 3.8. 

## 9. Selectivity 

**Figure 6:**
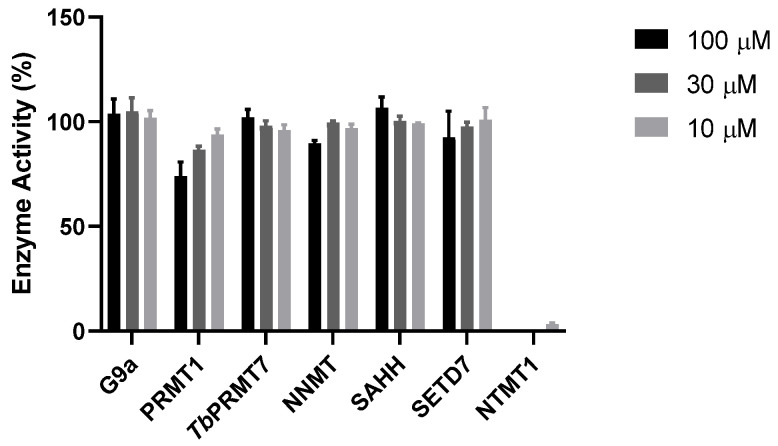
In-house selectivity studies of GD562. The compound was tested at 100, 30, and 10 µM (*n* = 2). Both SAM and substrate are at their *K*_m_ values.

Since GD562 exhibited the highest cellular inhibition activity and lower cytotoxicity among all tested peptidomimetic inhibitors (Table 2), the compound was chosen for in-house selectivity studies (Figure 6) by examining its inhibition activity against a panel of five other SAM-dependent protein methyltransferases and the coupling enzyme SAHH in a fluorescence assay. The results showed that GD562 did not inhibit over 50% of those enzyme activities at 100 μM, indicating that the compound was more than 100-fold selective for NTMT1 against other methyltransferases and SAHH. Furthermore, compared with DC541 [15], GD562 showed increased selectivity for NTMT1 over PRMT1. 

## 10. Discussion

In summary, a series of new peptidomimetic inhibitors were designed, synthesized, and evaluated based on the structures of previously reported inhibitors. We successfully optimized the cellular inhibition activity of the peptidomimetic inhibitor by introducing more hydrophobic function groups to replace the benzamide of DC541. The most cell-potent inhibitor GD562 exhibited over 100-fold selectivity over the other five SAM-dependent protein methyltransferases. Although GD562 showed decreased biochemical inhibition compared to DC541, it demonstrated an over 2-fold increase in cellular inhibition activity on me3-RCC1. In addition, it also inhibited the me3-SET with a similar IC_50_ as me3-RCC1. Besides, GD562 exhibited improved selectivity over DC541 regarding PRMT1. Noticeably, the cellular inhibition activity of NTMT1 by GD562 is not optimal since there is still a ~50-fold difference between its enzymatic and cellular IC_50_ values. Based on the PAMPA result, permeability alone did not explain the discrepancy between the biochemical and cellular IC_50_ values. Future directions include further improving the cellular potency of these compounds by adding substituent groups to the C-terminal phenyl ring, or testing new heterocycles at the C-region. Meanwhile, fluorescent labeling GD562 to monitor its cellular distribution would also be informative, as NTMT1 is predominantly located in the nucleus [1]. Nevertheless, GD562 is the first tetra-peptidomimetic inhibitor that can inhibit me3-RCC1 and me3-SET with an IC_50_ value of ~50 µM. Therefore, it can serve as a valuable tool to investigate the biological functions of NTMT1 in cells and as a new lead compound for further optimization. 

## Figures and Tables

**Table 1:**
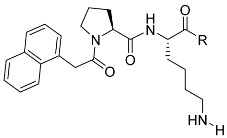
SAR of synthesized compounds with modifications at C-region.

ID	R	IC_50_ (μM)	cLogP	IC_50_/IC_50_ (DC541)
DC541	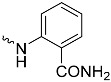	0.34 ± 0.03	0.7	1.0
1a (GD556)	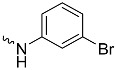	0.17 ± 0.008	2.9	0.50
1b (GD558)	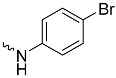	0.18 ± 0.007	2.9	0.53
1c (GD560)	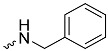	0.91 ± 0.03	1.7	2.7
1d (GD562)	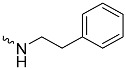	0.93 ± 0.04	1.9	2.7
1e (GD573)	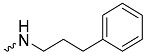	0.65 ± 0.01	2.3	1.9
1f (GD564)	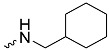	0.94 ± 0.03	2.4	2.8
1g (GD566)	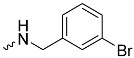	0.58 ± 0.03	2.5	1.7
1h (GD568)	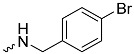	0.82 ± 0.07	2.5	2.4
1i (GD589)	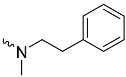	1.7 ± 0.07	2.3	5.0
1j (GD590)	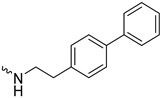	2.5 ± 0.09	3.8	7.4
1k (GD591)	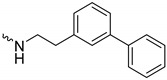	0.69 ± 0.08	3.8	2.0

N.D. = not determined. All experiments were performed in duplicates (*n* = 2) and presented as mean ± standard deviation (SD).

**Table 2:** Cellular N-terminal methylation inhibition, cytotoxicity, and permeability of compounds **1c**–**k**.

ID	GI_50_ (μM)	Cellular IC_50_ (μM)	Permeability (10^−6^ cm/s)
1c (GD560)	>300	<300	0.24 ± 0.06
1d (GD562)	>300	~50	0.26 ± 0.06
1e (GD573)	>300	~100	0.17 ± 0.08
1f (GD564)	>300	~100	0.47 ± 0.07
1g (GD566)	128 ± 20	<300	0.6 ± 0.1
1h (GD568)	135 ± 23	<300	0.9 ± 0.2
1j (GD590)	N.D	N.D.	0.9 ± 0.3
1k (GD591)	65 ± 7	N.D.	1.3 ± 0.4

Note: Verapamil was used as the positive control and was 8.6 ± 0.98 × 10**^−6^** cm/s in the PAMPA assay. All experiments were performed in duplicate (*n* = 2) and presented as mean ± SD. N.D. = not determined.

## Data Availability

Not applicable.

## Supplementary Materials

The following supporting information can be downloaded online. Table S1: MSλD Computed vs. Experimental Binding Free Energies (kcal/mol), Figure S1: Correlation between experimental binding affinities and average computed center of mass (COM) distances between C-region substituent rings and the phenyl ring of Tyr215. MS, NMR spectra, HRMS, and HPLC analysis of compounds **1a**–**k**.

## Abbreviations

NTMT, protein N-terminal methyltransferase; SAM, S-5′-adenosyl-L-methionine; SAH, S-5′-adenosyl-L-homocysteines; SAHH, SAH hydrolase; MT, methyltransferase; PKMT, protein lysine methyltransferase; PRMT, protein arginine methyltransferase; PRMT1, protein arginine methyltransferase 1; *Tb*PRMT7, *Trypanosoma brucei* protein arginine methyltransferase 7; G9a, euchromatic histone-lysine N-methyltransferase 2; SETD7, SET domain-containing protein 7; NNMT, nicotinamide N-methyltransferase; rt, room temperature; TFA, trifluoroacetic acid.
