# Supplementary material for: Improved Cell-Potent and Selective Peptidomimetic Inhibitors of Protein N-Terminal Methyltransferase 1
Source: Molecules. 2022 Feb 18;27(4):1381. doi: 10.3390/molecules27041381 (PMC8874984; doi:10.3390/molecules27041381)
Supplement: Supplementary file 1 [file molecules-27-01381-s001.zip › molecules-1543163-supplementary.pdf]

Supporting Information

Improved Cell-Potent and Selective Peptidomimetic Inhibitors of Protein N-Terminal Methyltransferase 1

Guangping Dong [1], Iredia D. Iyamu [1], Jonah Z. Vilseck [2], Dongxing Chen [1] and Rong Huang [1,*]

[1] Department of Medicinal Chemistry and Molecular Pharmacology, Purdue Institute for Drug Discovery, Purdue University Center for Cancer Research, Purdue University, West Lafayette, IN 47907, USA; dong216@purdue.edu (G.D.); iiyamu@purdue.edu (I.D.I.); chendoxi@hotmail.com (D.C.)

[2] Department of Biochemistry and Molecular Biology, Center for Computational Biology and Bioinformatics, Indiana University School of Medicine, Indianapolis, IN 46202, USA; jvilseck@iu.edu

* Correspondence: huang-r@purdue.edu; Tel.: +1(765)494-3426

Table of Contents

A. Experimental section
B. MS characterization of compounds **1a**-**k**.
C. MS and HPLC analysis of compounds **1a**-**k**.
D. NMR spectra of compounds **1a**-**k**.
E. Supplemental Table S1 and Figure S1
F. References

Experimental section

Protein expression and purification. Expression and purification of NTMT1, G9a, SETD7, PRMT1, PRMT3, NNMT, and *Tb*PRMT7 was performed as previously described [1–7].

NTMT1 biochemical assays. A fluorescence-based SAHH-coupled assay was applied to study the IC_{50} values for all the compounds. The assay was performed under the following conditions in a final well volume of 40 μL: 5 mM Tris (pH = 7.5), 50 mM KCl, 0.01% Triton X-100, 5 μM SAHH, 0.2 μM NTMT1, 100 μM SAM, and 15 μM ThioGlo1; or 25 mM Tris (pH 7.5), 50 mM KCl, 0.01% Triton X-100, 10μM SAHH, 0.1μM NTMT1, 100 μM SAM, and 10 μM ThioGlo4. The inhibitors were added at concentrations ranging from 0.15 nM to 10 μM.

After 10 min incubation, reactions were initiated by the addition of 0.5 μM GPKRIA peptide. Fluorescence was monitored on a BMG ClariOtar microplate reader with excitation 370 nm and emission 500 nm (for Thioglo 1), or excitation 400 nm and emission 465 nm (for Thioglo 4). Data were processed by using GraphPad Prism software 8.0. For the inhibition mechanism studies were performed as previously described [6].

Selectivity assays. The selectivity studies of G9a, SETD7, PRMT1, NNMT, and *Tb*PRMT7 were performed as previously described [8,9].

Cellular N-methylation level. HCT116 cells were seeded 40,000 cells/well on 24-well plates in the presence of 1x PBS or DC541 at different concentrations for 3 days. Then cells were lysed in 1x RIPA cell lysis buffer (25 mM Tris-HCl, pH 7.6, 150 mM NaCl, 1% NP-40, 1% sodium deoxycholate, 0.1% SDS, protease inhibitors) and incubated for 30 min on ice. The cell lysates were centrifuged at 15,000 rpm for 10 min and the precipitates were removed. The concentration of total protein was quantified by bicinchoninic acid (BCA) protein assay kit (ThermoFisher, #23228). Equal amounts of total protein were mixed with 4x loading dye and loaded onto a 12.5% SDS–PAGE gel and separated. The gel was transferred to a polyvinylidene difluoride (PVDF) membrane using the BioRad Trans-Blot Turbo system. The membrane was then blocked for 1 h in 5% milk TBST solution and washed with 1x TBST solution three times. The membrane was incubated with the anti-me3-SPK antibody at 4 °C for 12 h, washed with 1x TBST solution three times, and then incubated with Rabbit IgG-HRP antibody (Cell Signaling, #7074S) for 1 h at room temperature. The membrane was washed with 1x TBST solution three times and detected using a Protein Simple FluorChem imaging system. Image quantification was done using ImageJ software (NIH). All bands were compared to the respective untreated control, which was set at 1.0.

Cell viability assay. HCT116 cells were seeded as 5,000 cells/well on 96- well plates in the presence of 1x PBS or DC541 at different concentrations for 24, 48, or 72 hours. Cell viability was assessed using 0.2 mg/ml resazurin solutions prepared from resazurin sodium salt (Acros Organics™, AC418900050) dissolved in sterile 1 x PBS. Then, the cells were incubated with 10 μl resazurin solution (10% of cell culture volume) for four hours at 37 °C. The fluorescence was measured using a CLARIOstar microplate reader (Ex = 540 nm, Em = 620 nm) at 37 °C. Cell viability was calculated as 100% × (fluorescence of treated cells – fluorescence of background controls) / (fluorescence of 1x PBS controls – fluorescence of background controls).

Molecular Modeling. Multisite λ-dynamics calculations were run in the CHARMM molecular simulation package at 25° C and 1 atm in the isothermal-isobaric ensemble [10,11]. Initial starting coordinates were obtained from the crystal structure of DC113 bound to NTMT1 (PDBID: 7K3D) [9]. Ligands were manually built in UCSF Chimera [12]. Protein and ligand components were represented with CHARMM36 and CGenFF force field parameters [13-17], respectively, and the complex was solvated within a cubic box of TIP3P water molecules with the CHARMM-GUI [18,19]. Enough Na^+ and Cl^- ions were added to neutralize the system and provide an ionic concentration of 0.1 M. Long range interactions were smoothed to zero with force switching between 10.0 – 12.0 Å and soft-core non-bonded potentials were used to avoid endpoint singularities for alchemical sampling [20,21]. Prior to MSλD production sampling, the system was minimized with 500 steps of steepest descent minimization, and λ-biases were determined over a total of 153 ns of sampling with the Adaptive Landscape Flattening algorithm [21]. Five replicate MSλD production runs of 25 ns each were performed to determine the final relative free energy differences. Relative free energies of binding were then converted into absolute values for easier comparison to experiment [22]. Figures were made with PyMOL [23].

Parallel Artificial Membrane Permeability Assay [24,25]. The PAMPA assay was performed with Corning BioCoat pre-coated PAMPA plate system (Corning 353015). The stock solutions in 10 mM DMSO was diluted with PBS to a final concentration of 200 μM with 2% DMSO (v/v). Verapamil was added as a control. The pre-coated PAMPA plate system was warmed to room temperature and the donor plate (bottom) was filled with 300 μL of the diluted sample solution while the acceptor plate (top) was filled with 200 μL of the buffer. The acceptor plate was slowly lowered into the receiver plate and the assembly was incubated at room temperature for 5 h. The plate sandwich was separated and the concentration of the sample in both the donor and receiver plate was determined by UV spectrometry using CLARIOstar plate reader set at 254 and 280 nM. The permeability of each compound was calculated using the formula:

$$P_e = \frac{-In[1 - C_A(t)/C_{equilibrium}]}{A * (1/V_D + 1/V_A) * t}$$

$$C_{equilibrium} = [C_D(t) * V_D + C_A(t) * V_A]/(A * (V_D + V_A))$$

Where

C_D (t) = compound concentration in donor well at time t.

C_A (t) = compound concentration in acceptor well at time t.

V_D = donor well volume

V_A = acceptor well volume

A = filter area

t = incubation time

MS characterization of compounds.

Compound **1a (GD556)**. MALDI-MS (positive) m/z: calcd for $C_{29}H_{33}BrN_4O_3$ $[M + H]^+$ m/z 567.1716, found m/z 567.3305.

Compound **1b** (**GD558)**. MALDI-MS (positive) m/z: calcd for $C_{29}H_{33}BrN_4O_3$ $[M + H]^+$ m/z 567.1716, found m/z 567.3351.

Compound **1c** (**GD560)**. MALDI-MS (positive) m/z: calcd for $C_{30}H_{36}N_4O_3$ $[M + H]^+$ m/z 501.2821, found m/z 501.4417.

Compound **1d** (**GD562)**. MALDI-MS (positive) m/z: calcd for $C_{31}H_{38}N_4O_3$ $[M + H]^+$ m/z 515.2944, found m/z 501.4635.

Compound **1e** (**GD573)**. MALDI-MS (positive) m/z: calcd for $C_{32}H_{40}N_4O_3$ $[M + H]^+$ m/z 529.3100, found m/z 529.4910.

Compound **1f** (**GD564)**. MALDI-MS (positive) m/z: calcd for $C_{30}H_{42}N_4O_3$ $[M + H]^+$ m/z 507.3257, found m/z 507.4806.

Compound **1g** (**GD566)**. MALDI-MS (positive) m/z: calcd for $C_{30}H_{35}BrN_4O_3$ $[M + H]^+$ m/z 579.1893, found m/z 579.3701.

Compound **1h** (**GD568)**. MALDI-MS (positive) m/z: calcd for $C_{30}H_{35}BrN_4O_3$ $[M + H]^+$ m/z 581.1872, found m/z 581.3701.

Compound **1i** (**GD589)**. MALDI-MS (positive) m/z: calcd for $C_{31}H_{38}N_4O_3$ $[M + H]^+$ m/z

529.2944, found m/z 529.4852.

Compound **1j** (**GD590**). MALDI-MS (positive) m/z: calcd for $C_{37}H_{42}N_4O_3$ $[M + H]^+$ m/z 591.3257, found m/z 591.5327.

Compound **1k** (**GD591**). MALDI-MS (positive) m/z: calcd for $C_{37}H_{42}N_4O_3$ $[M + H]^+$ m/z 591.3257, found m/z 591.5327.

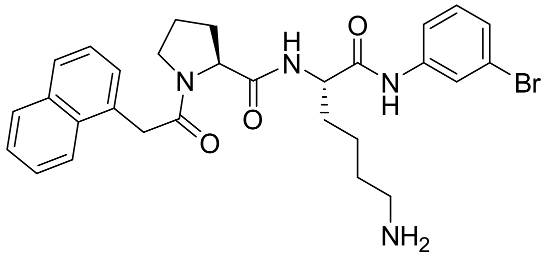

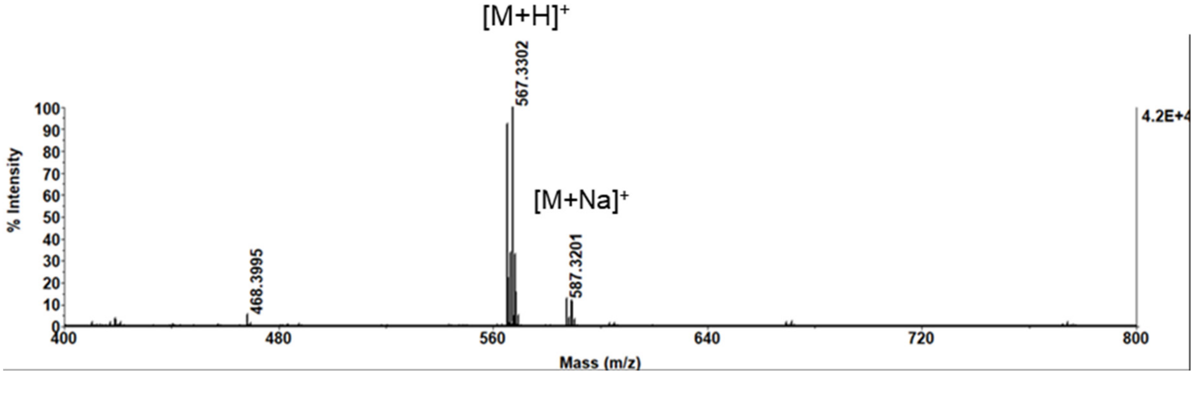

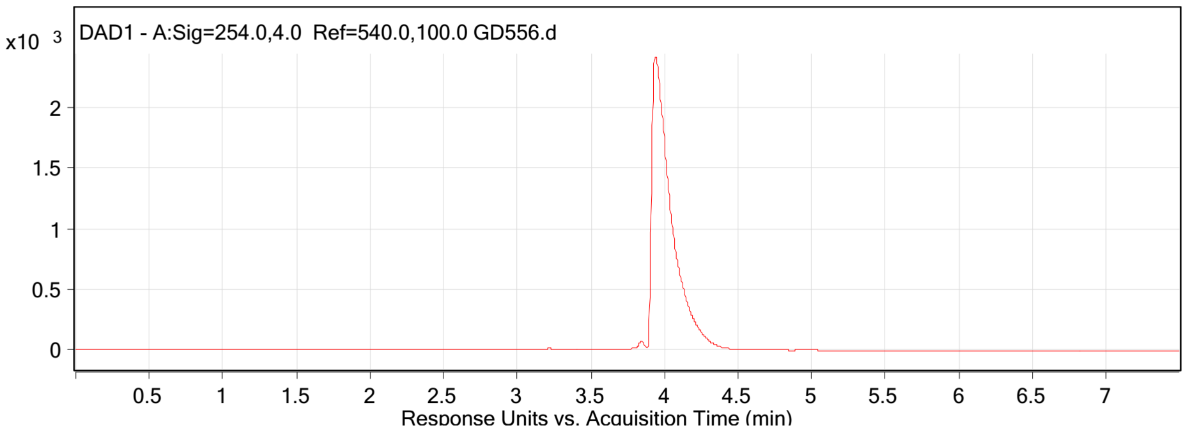
DAD1 - A:Sig=254.0,4.0 Ref=540.0,100.0 GD556.d
x10 3
Response Units vs. Acquisition Time (min)

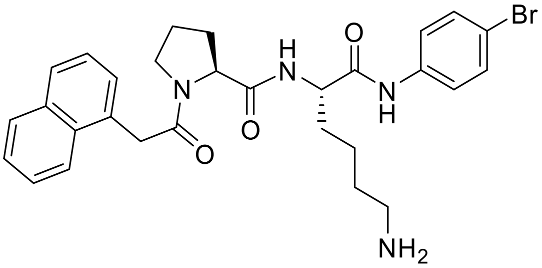

1b

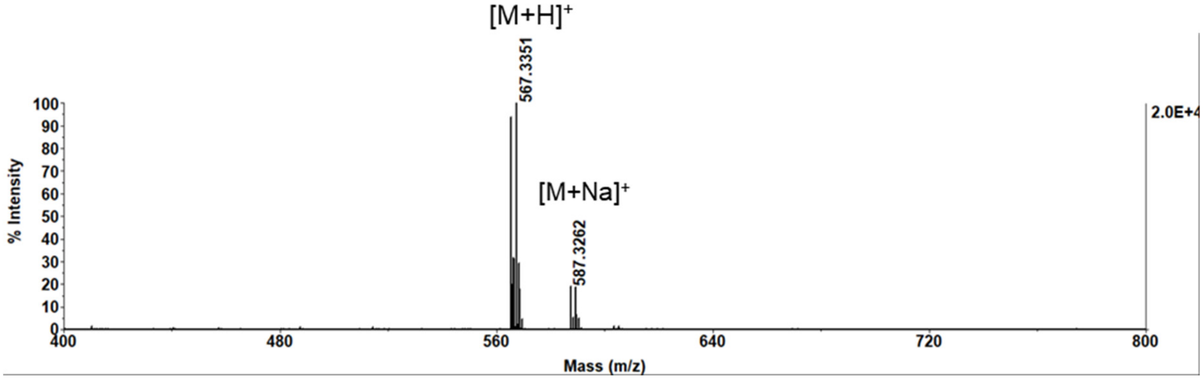
[M+H]+
567.3351
[M+Na]+
587.3262
% Intensity
Mass (m/z)
2.0E+4

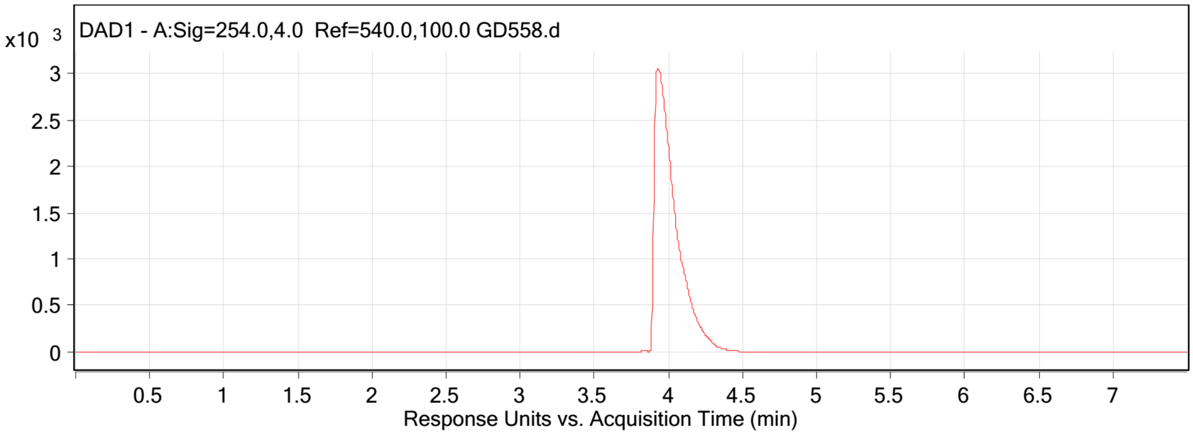

x10 3
DAD1 - A:Sig=254.0,4.0 Ref=540.0,100.0 GD558.d
Response Units vs. Acquisition Time (min)

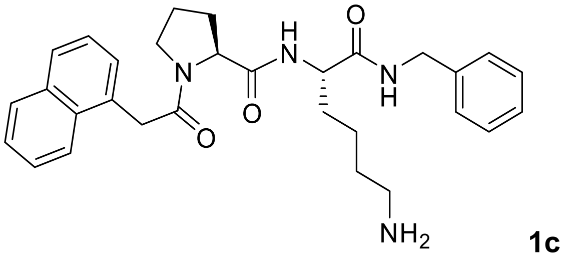

1c

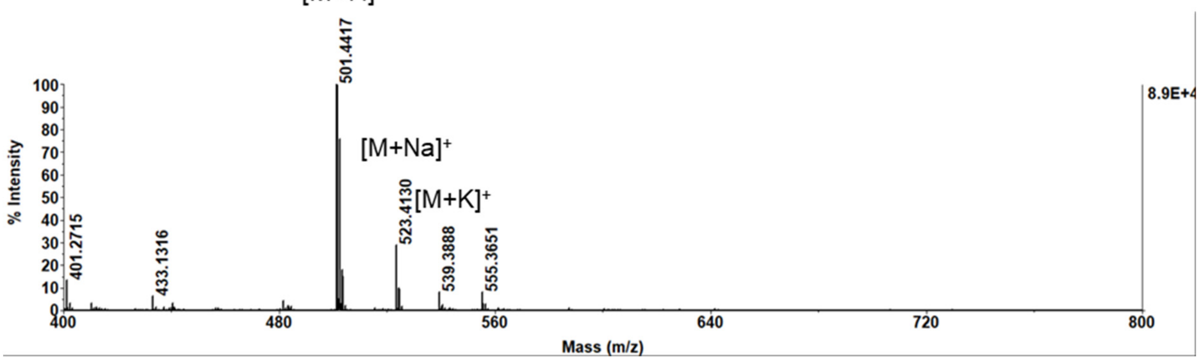

[M+H]+
501.4417
[M+Na]+
523.4130
[M+K]+
401.2715
433.1316
539.3888
555.3651
8.9E+4
% Intensity
Mass (m/z)

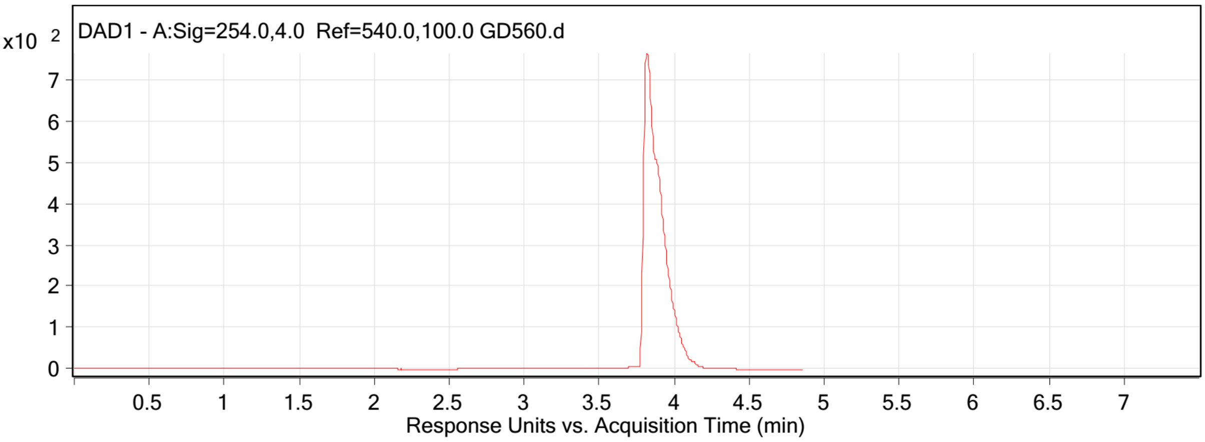
x10 2
DAD1 - A:Sig=254.0,4.0 Ref=540.0,100.0 GD560.d
Response Units vs. Acquisition Time (min)

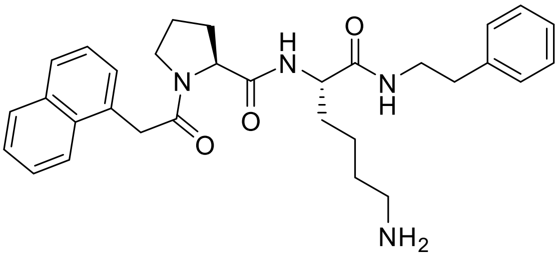

1d

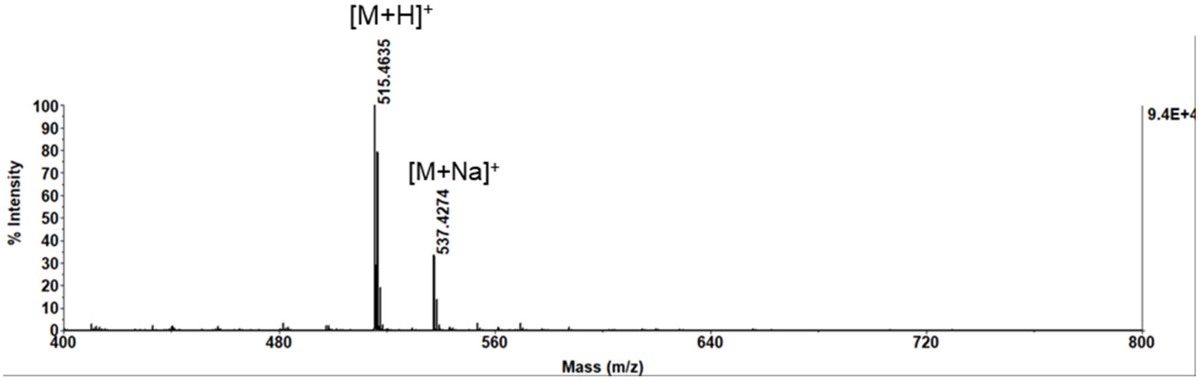
[M+H]+
515.4635
[M+Na]+
537.4274
9.4E+4
% Intensity
Mass (m/z)

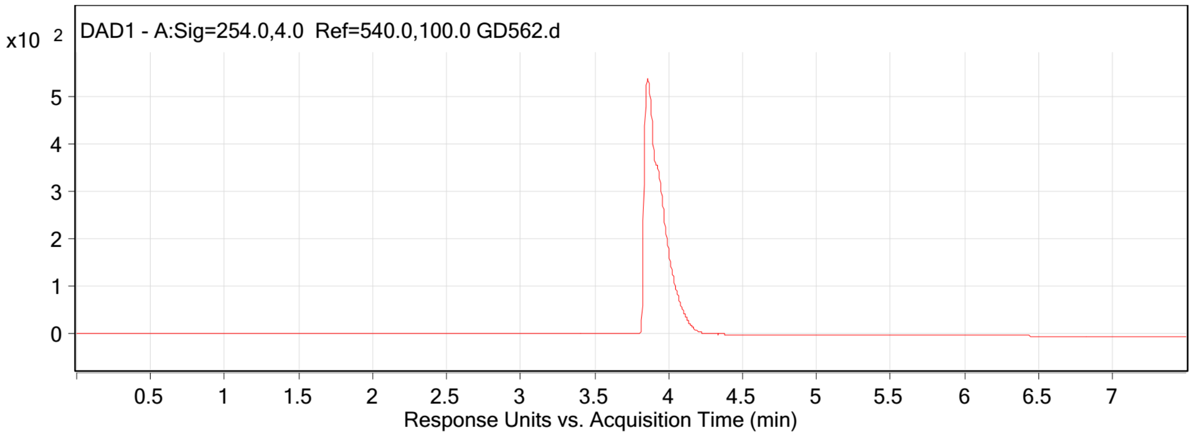

x10 2
DAD1 - A:Sig=254.0,4.0 Ref=540.0,100.0 GD562.d
Response Units vs. Acquisition Time (min)

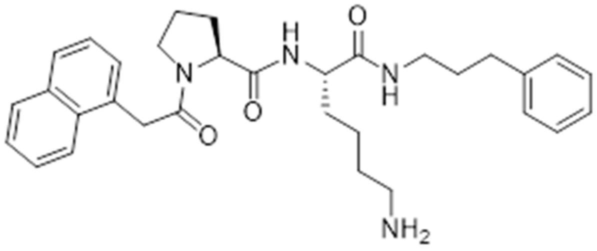

1e

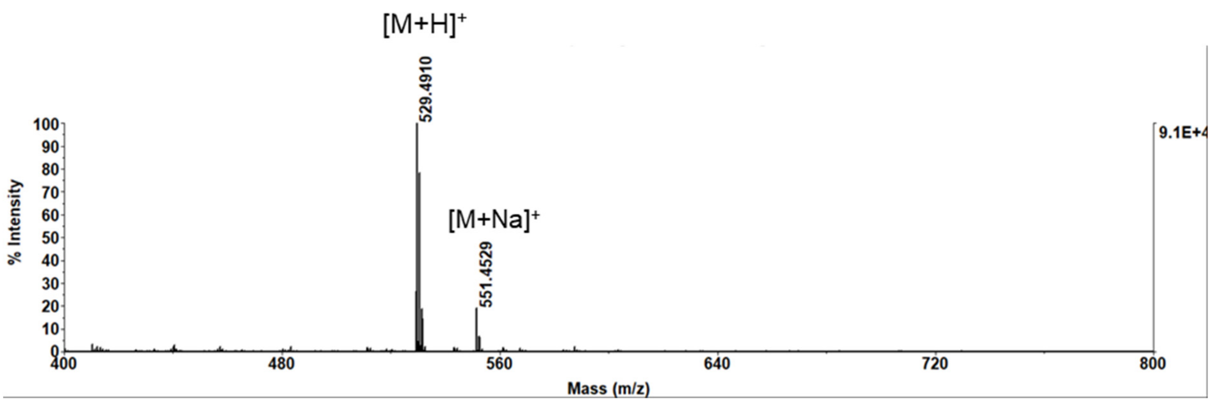

[M+H]+
529.4910
[M+Na]+
551.4529
9.1E+4
% Intensity
Mass (m/z)

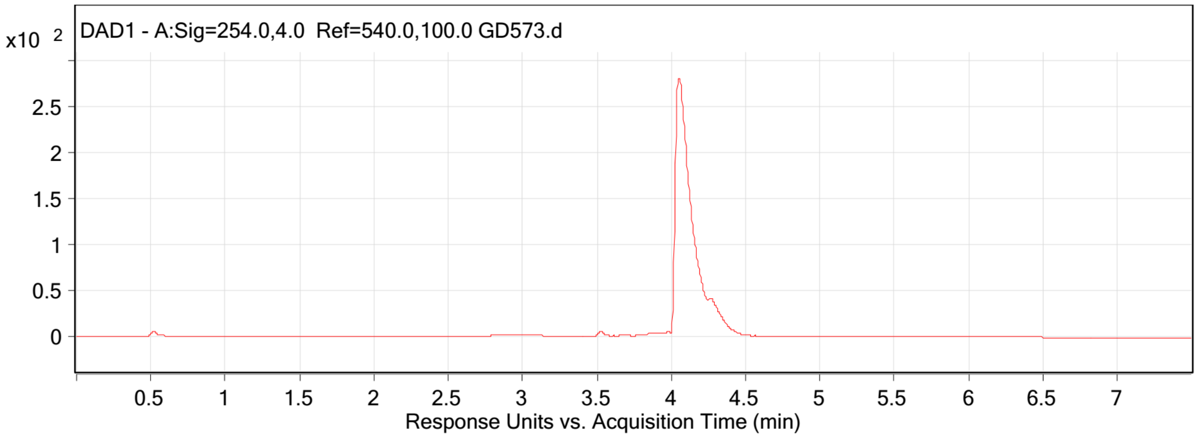

x10 2
DAD1 - A:Sig=254.0,4.0 Ref=540.0,100.0 GD573.d
2.5
2
1.5
1
0.5
0
0.5
1
1.5
2
2.5
3
3.5
4
4.5
5
5.5
6
6.5
7
Response Units vs. Acquisition Time (min)

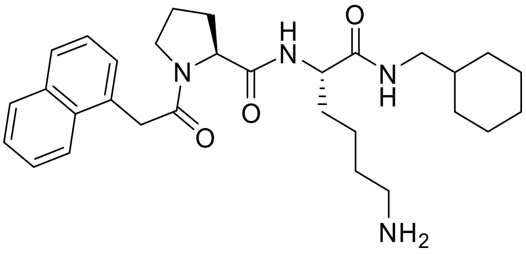

H
N
O
N
O
O
N
H
NH2

1f

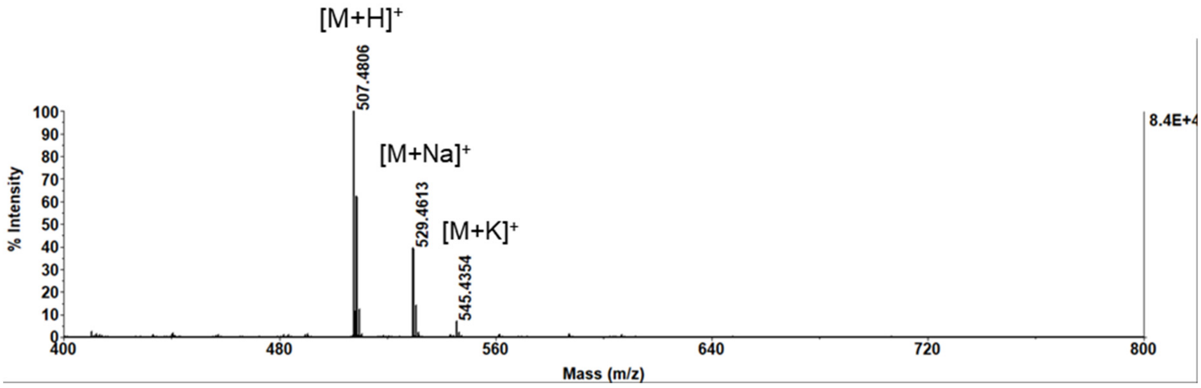

[M+H]+
507.4806
[M+Na]+
529.4613
[M+K]+
545.4354
8.4E+4
% Intensity
100
90
80
70
60
50
40
30
20
10
0
400
480
560
640
720
800
Mass (m/z)

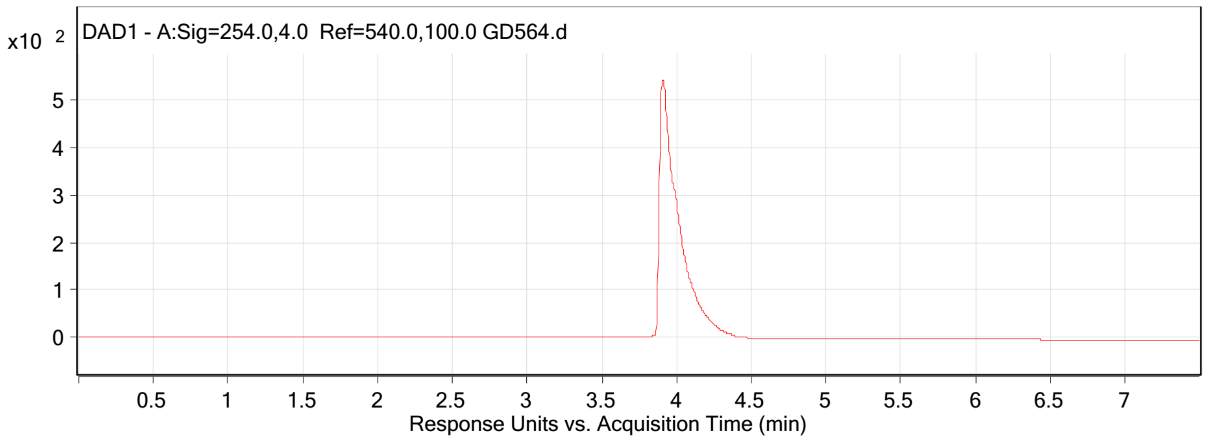

x10 2
DAD1 - A:Sig=254.0,4.0 Ref=540.0,100.0 GD564.d
Response Units vs. Acquisition Time (min)

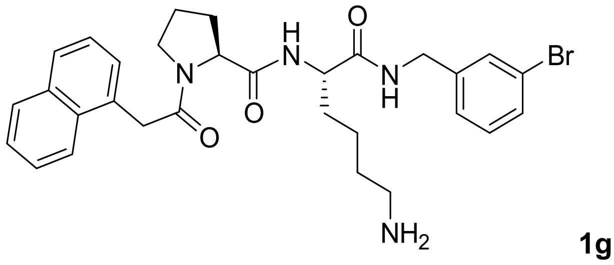

Br
NH2

1g

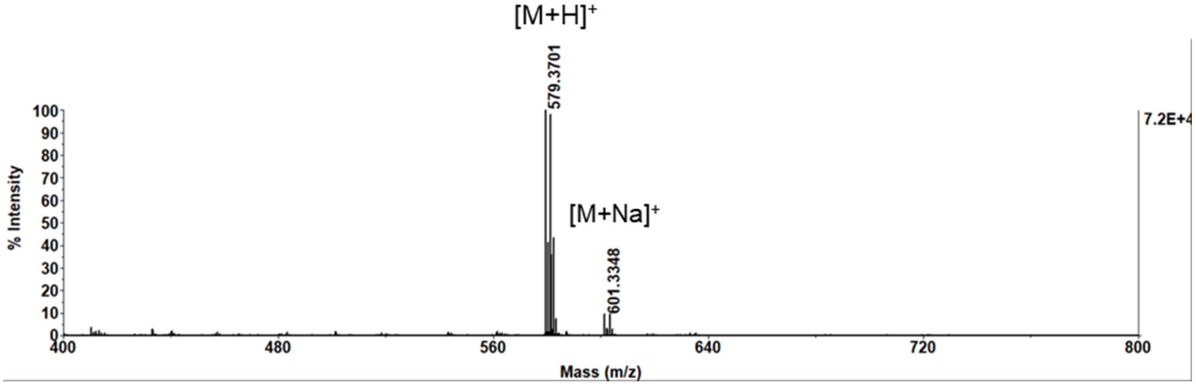

[M+H]+
579.3701
[M+Na]+
601.3348
7.2E+4
% Intensity
Mass (m/z)

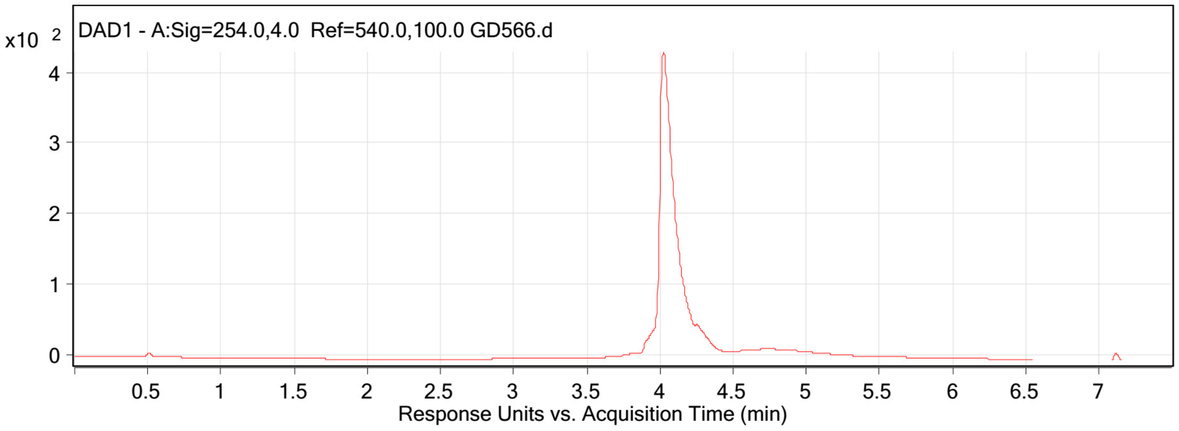
x10 2
DAD1 - A:Sig=254.0,4.0 Ref=540.0,100.0 GD566.d
Response Units vs. Acquisition Time (min)

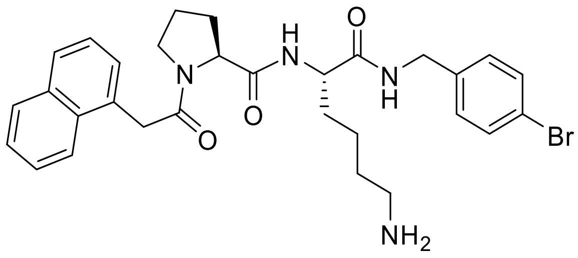

1h

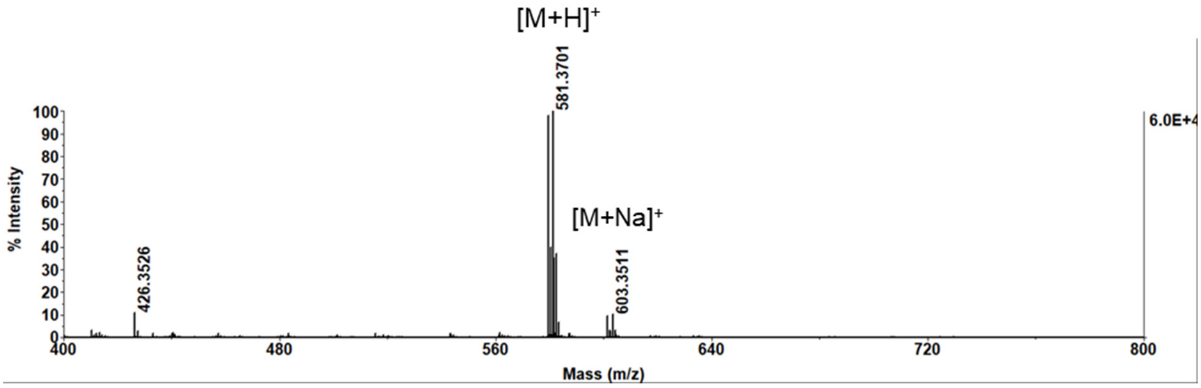
[M+H]+
581.3701
[M+Na]+
603.3511
426.3526
6.0E+4
% Intensity
Mass (m/z)

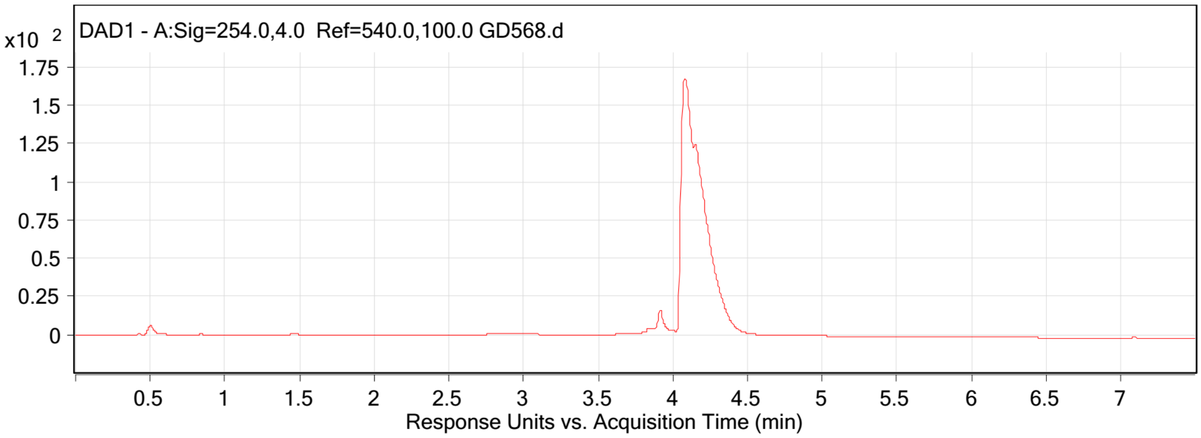
DAD1 - A:Sig=254.0,4.0 Ref=540.0,100.0 GD568.d
x10 2
1.75
1.5
1.25
1
0.75
0.5
0.25
0
0.5 1 1.5 2 2.5 3 3.5 4 4.5 5 5.5 6 6.5 7
Response Units vs. Acquisition Time (min)

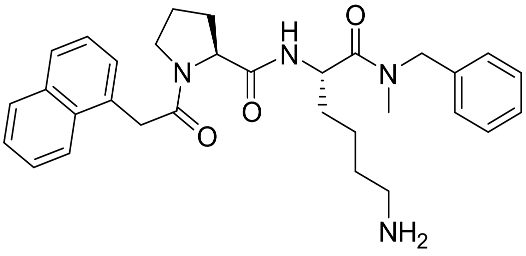
H
N
O
N
O
O
N
NH2

1i

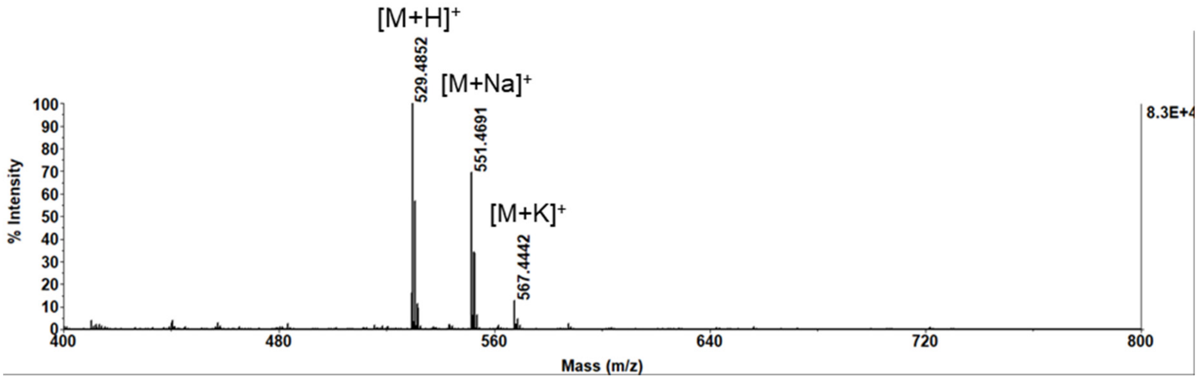
[M+H]+
529.4852
[M+Na]+
551.4691
[M+K]+
567.4442
8.3E+4
% Intensity
100
90
80
70
60
50
40
30
20
10
0
400 480 560 640 720 800
Mass (m/z)

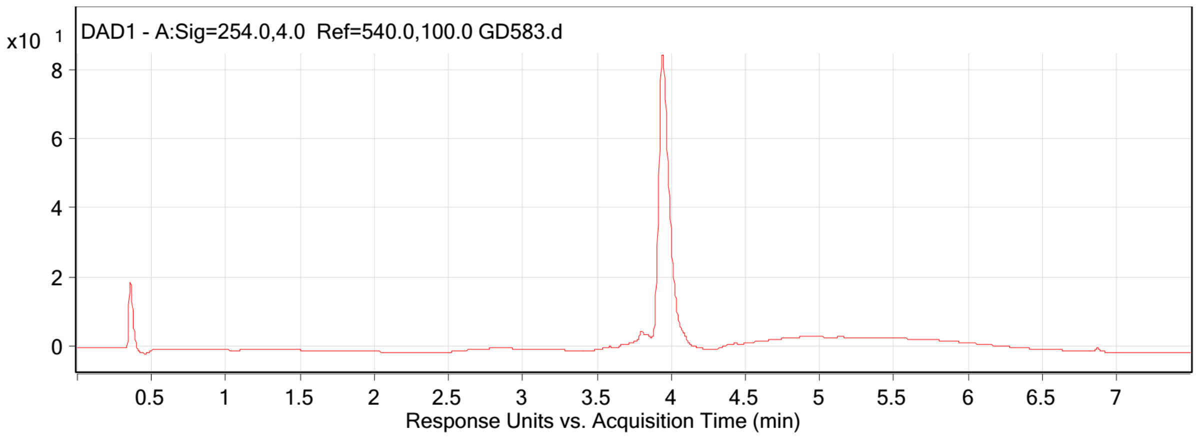

x10 1
DAD1 - A:Sig=254.0,4.0 Ref=540.0,100.0 GD583.d
Response Units vs. Acquisition Time (min)

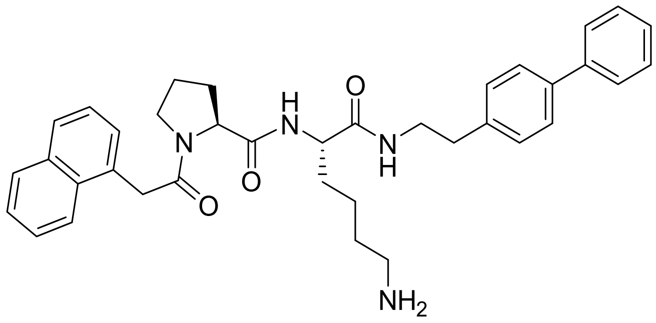

N
H
N
O
O
N
H
O
NH2

1j

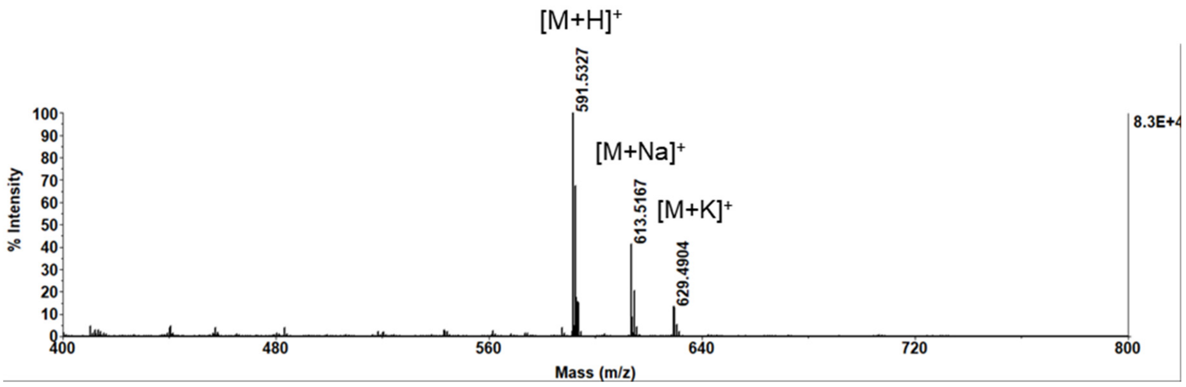

[M+H]+
591.5327
[M+Na]+
613.5167
[M+K]+
629.4904
8.3E+4
% Intensity
Mass (m/z)

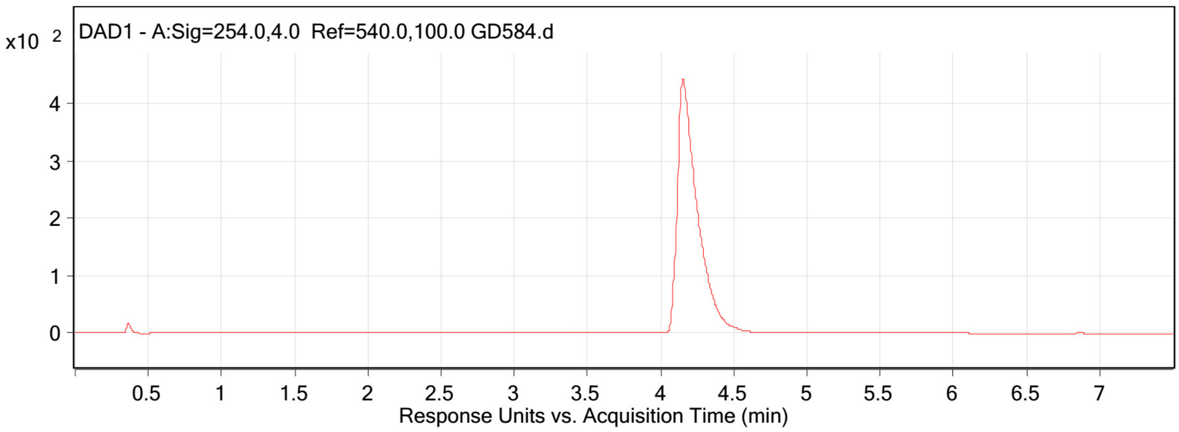

x10 2
DAD1 - A:Sig=254.0,4.0 Ref=540.0,100.0 GD584.d
4
3
2
1
0
0.5
1
1.5
2
2.5
3
3.5
4
4.5
5
5.5
6
6.5
7
Response Units vs. Acquisition Time (min)

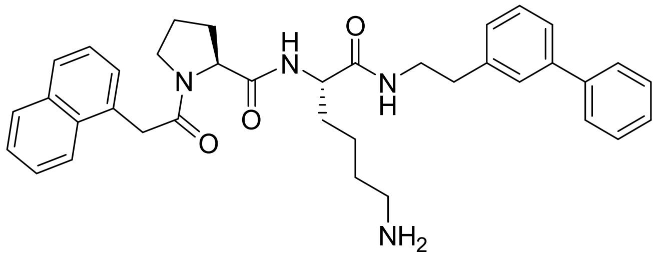

N
H
N
O
O
O
N
H
NH2

1k

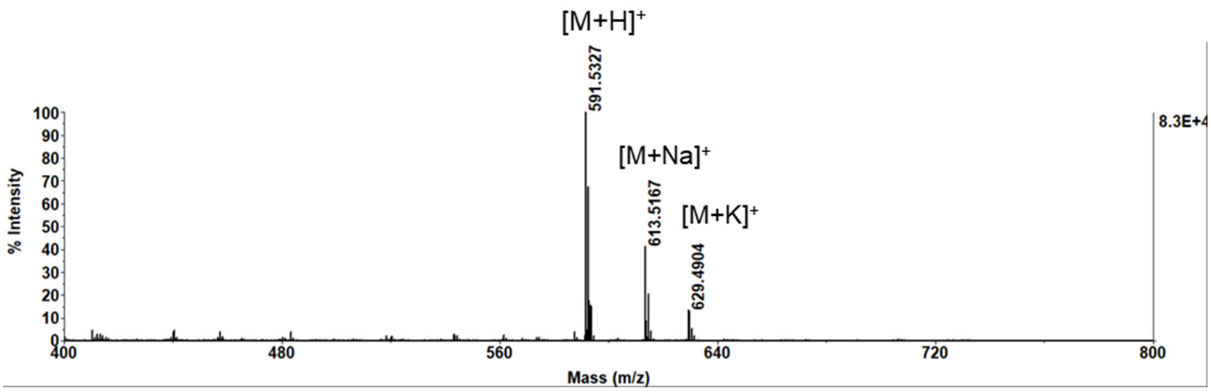

[M+H]+
591.5327
[M+Na]+
613.5167
[M+K]+
629.4904
8.3E+4
% Intensity
100
90
80
70
60
50
40
30
20
10
0
400
480
560
640
720
800
Mass (m/z)

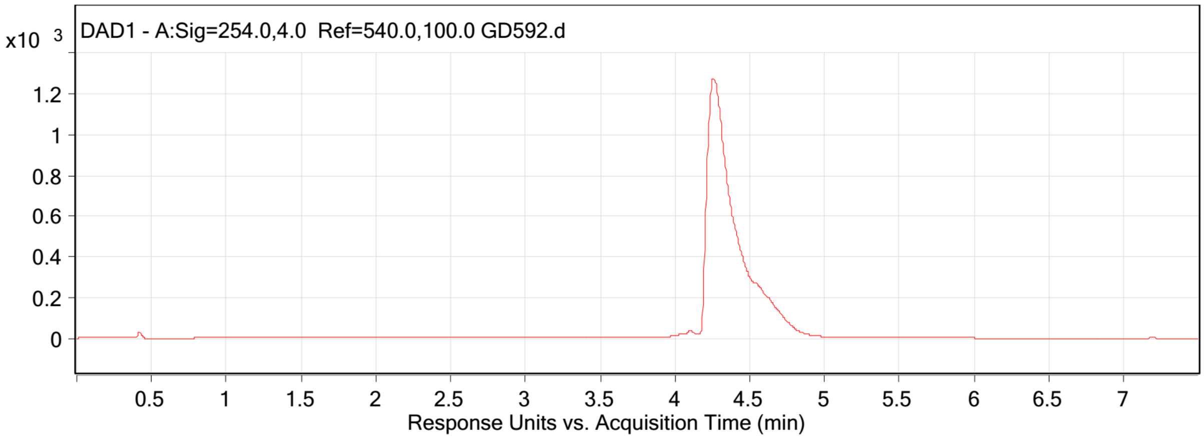

NMR spectra of compounds 1a-k.

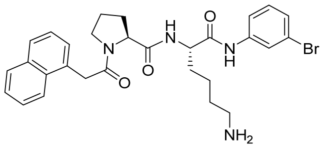

1a

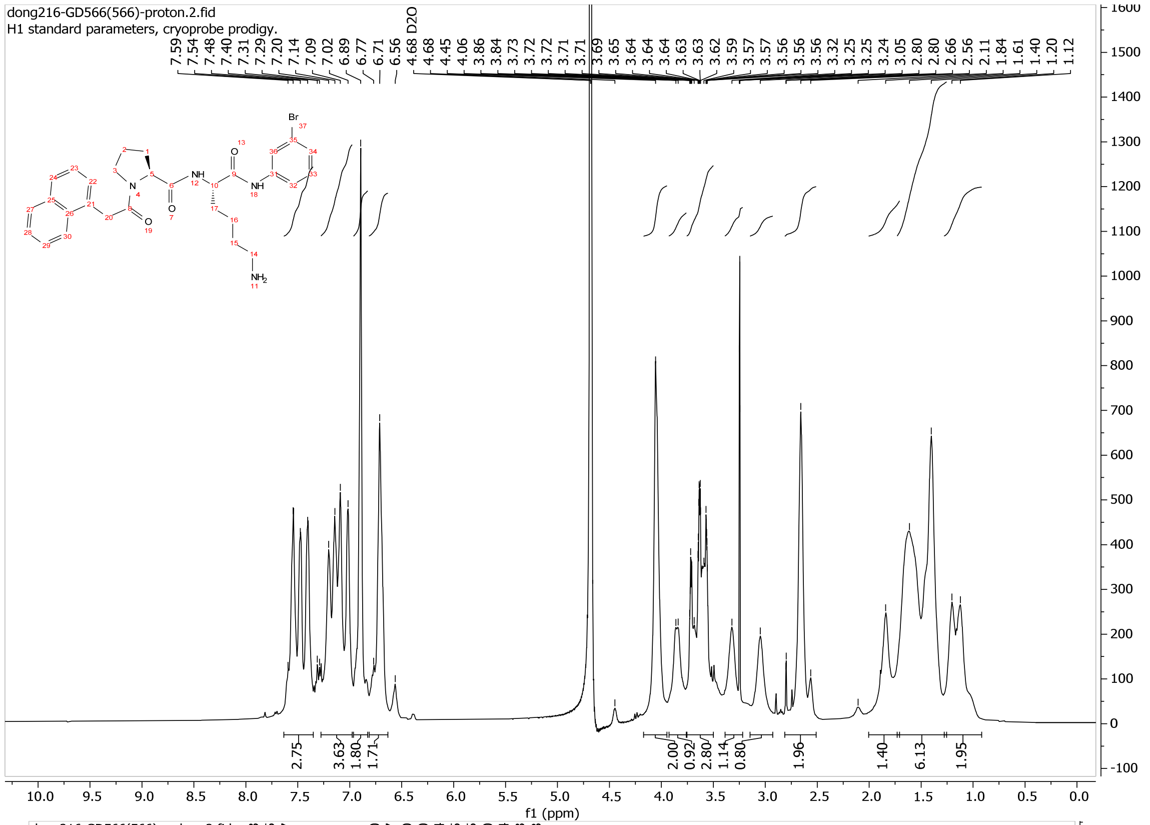
dong216-GD566(566)-proton.2.fid
H1 standard parameters, cryoprobe prodigy.
7.59 7.54 7.48 7.40 7.31 7.29 7.20 7.14 7.09 7.02 6.89 6.77 6.71 6.56 4.68 D2O 4.68 4.45 4.06 3.86 3.84 3.73 3.72 3.72 3.71 3.71 3.69 3.65 3.64 3.64 3.64 3.63 3.63 3.62 3.59 3.57 3.57 3.56 3.56 3.56 3.32 3.25 3.25 3.24 3.05 2.80 2.80 2.66 2.56 2.11 1.84 1.61 1.40 1.20 1.12
2.75 3.63 1.80 1.71 2.00 0.92 2.80 1.14 0.80 1.96 1.40 6.13 1.95
f1 (ppm)
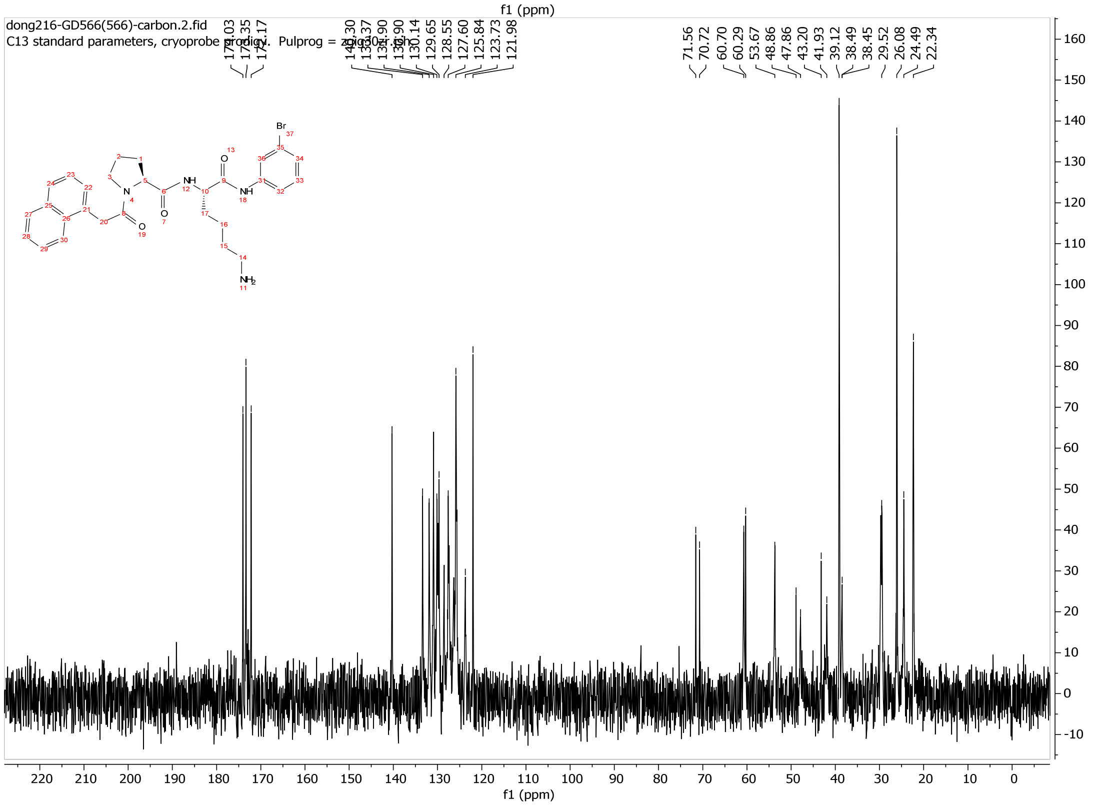
dong216-GD566(566)-carbon.2.fid
C13 standard parameters, cryoprobe prodigy.
174.03 173.35 172.17 140.30 133.37 131.90 130.90 130.14 129.65 128.55 127.60 125.84 123.73 121.98 71.56 70.72 60.70 60.29 53.67 48.86 47.86 43.20 41.93 39.12 38.49 38.45 29.52 26.08 24.49 22.34
f1 (ppm)
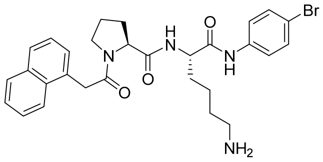

1b

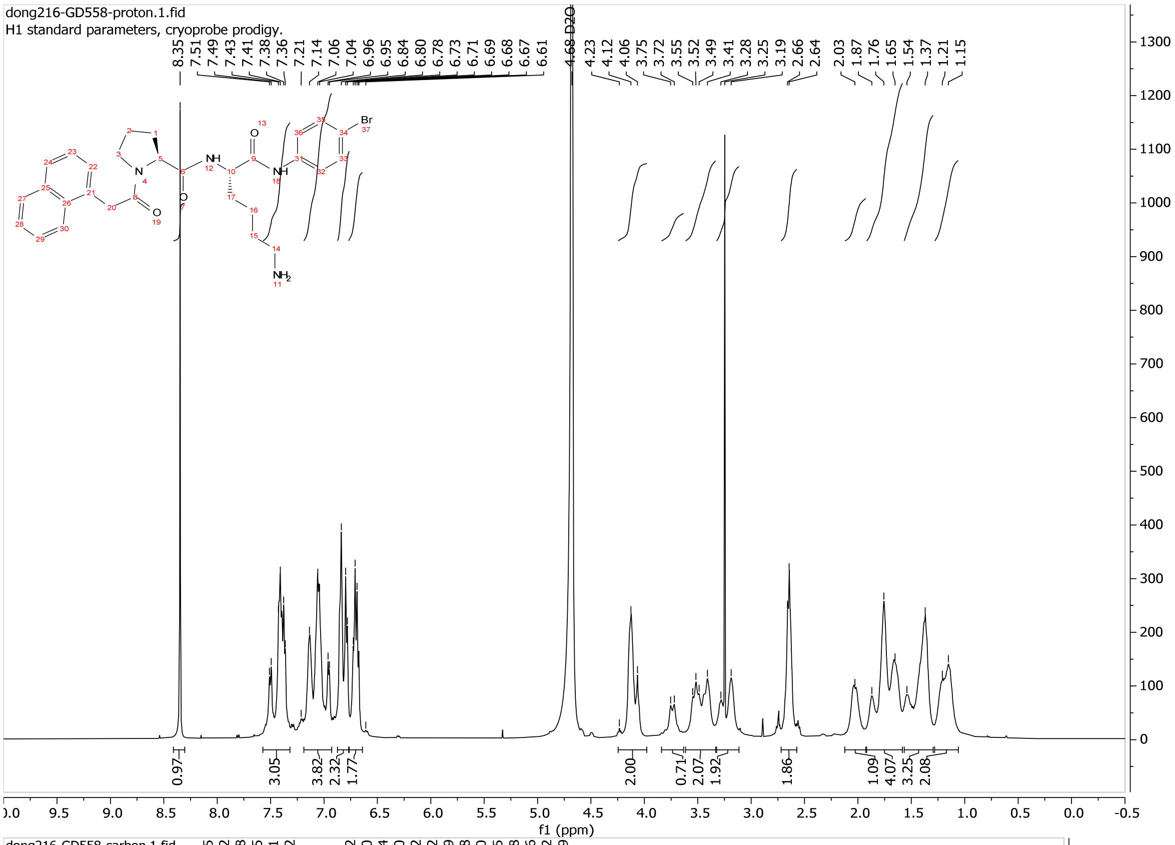
dong216-GD558-proton.1.fid
H1 standard parameters, cryoprobe prodigy.
8.35 7.51 7.49 7.43 7.41 7.38 7.36 7.21 7.14 7.06 7.04 6.96 6.95 6.84 6.80 6.78 6.73 6.71 6.69 6.68 6.67 6.61
4.68 D2O
4.23 4.12 4.06 3.75 3.72 3.55 3.52 3.49 3.41 3.28 3.25 3.19 2.66 2.64 2.03 1.87 1.76 1.65 1.54 1.37 1.21 1.15
0.97 3.05 3.82 2.32 1.77 2.00 0.71 2.07 1.92 1.86 1.09 4.07 3.25 2.08
f1 (ppm)

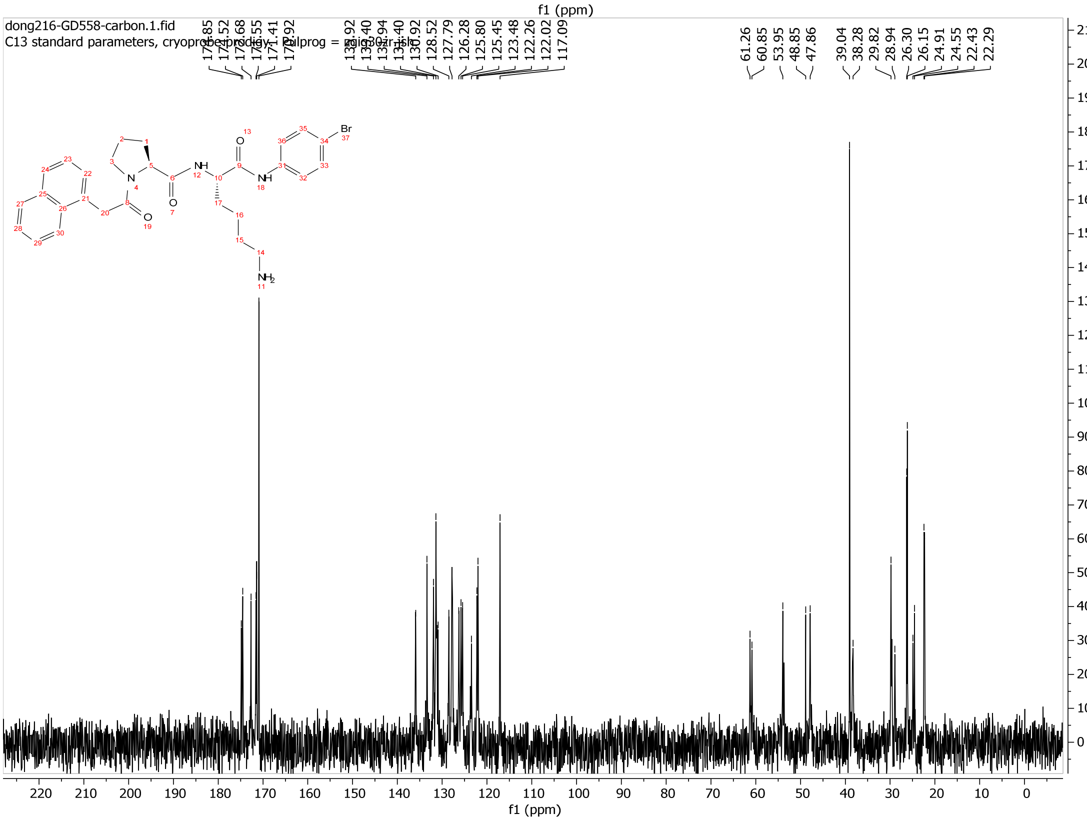
dong216-GD558-carbon.1.fid
C13 standard parameters, cryoprobe prodigy. Pulprog = zgig30zr.ish
130.92 128.52 127.79 126.28 125.80 125.45 123.48 122.26 122.02 117.09
61.26 60.85 53.95 48.85 47.86 39.04 38.28 29.82 28.94 26.30 26.15 24.91 24.55 22.43 22.29
f1 (ppm)

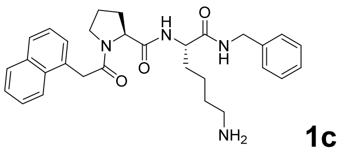

1c

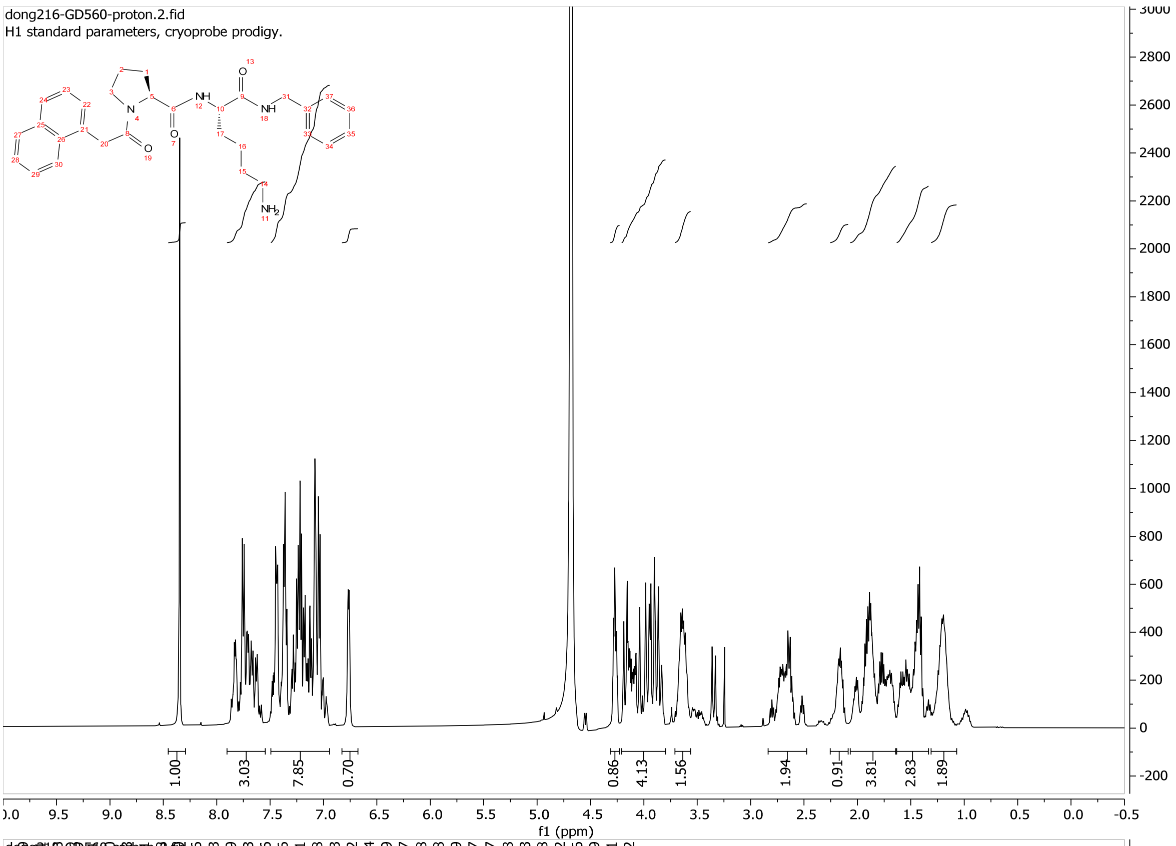
dong216-GD560-proton.2.fid
H1 standard parameters, cryoprobe prodigy.
1.00
3.03
7.85
0.70
0.86
4.13
1.56
1.94
0.91
3.81
2.83
1.89
f1 (ppm)

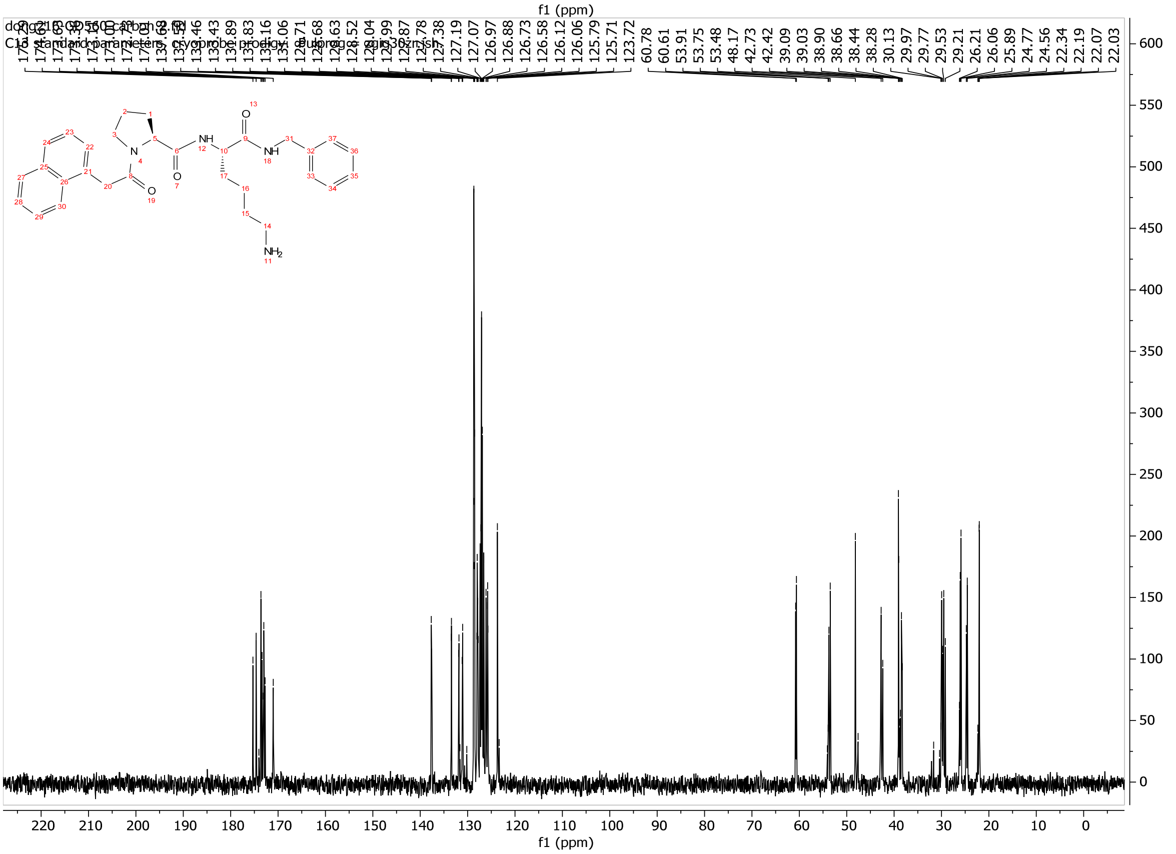
127.19
127.07
126.97
126.88
126.73
126.58
126.12
126.06
125.79
125.71
123.72
60.78
60.61
53.91
53.75
53.48
48.17
42.73
42.42
39.09
39.03
38.90
38.66
38.44
38.28
30.13
29.97
29.77
29.53
29.21
26.21
26.06
25.89
24.77
24.56
22.34
22.19
22.07
22.03
f1 (ppm)

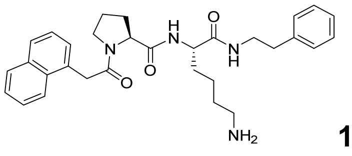

1d

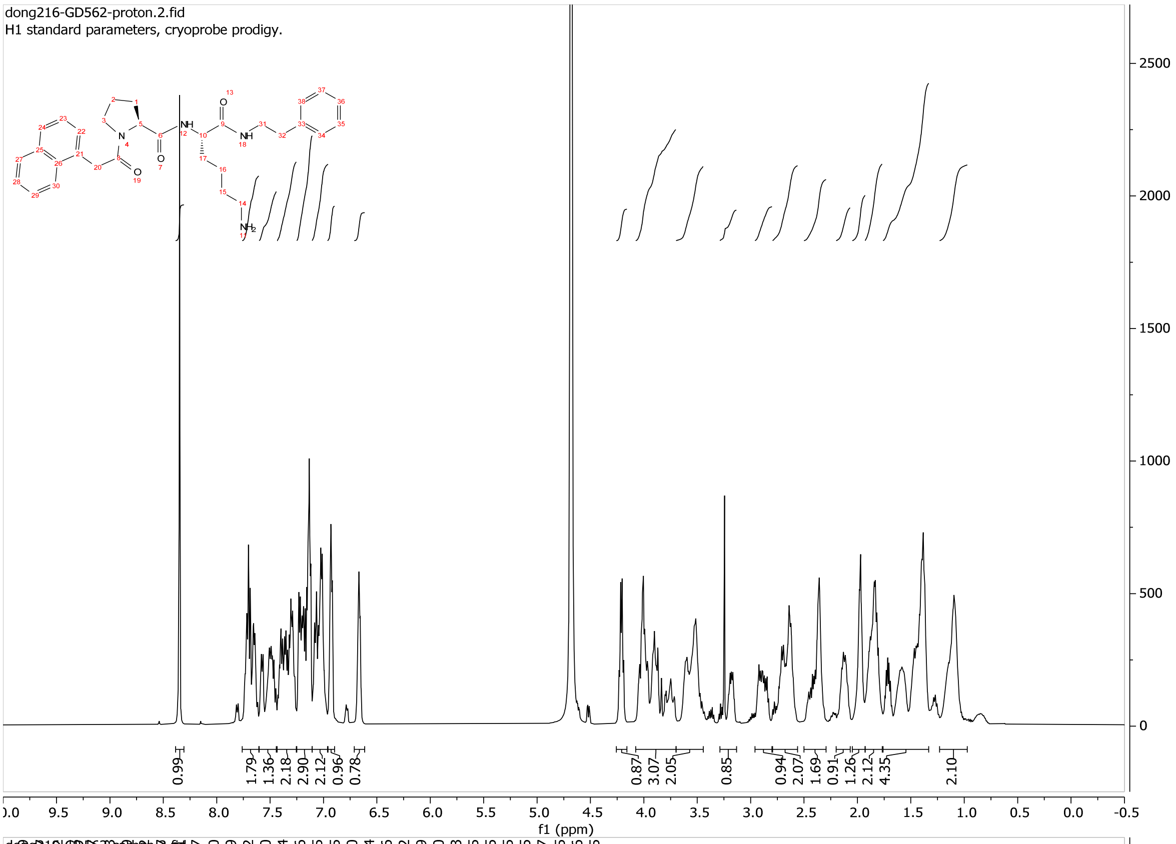
dong216-GD562-proton.2.fid
H1 standard parameters, cryoprobe prodigy.
f1 (ppm)

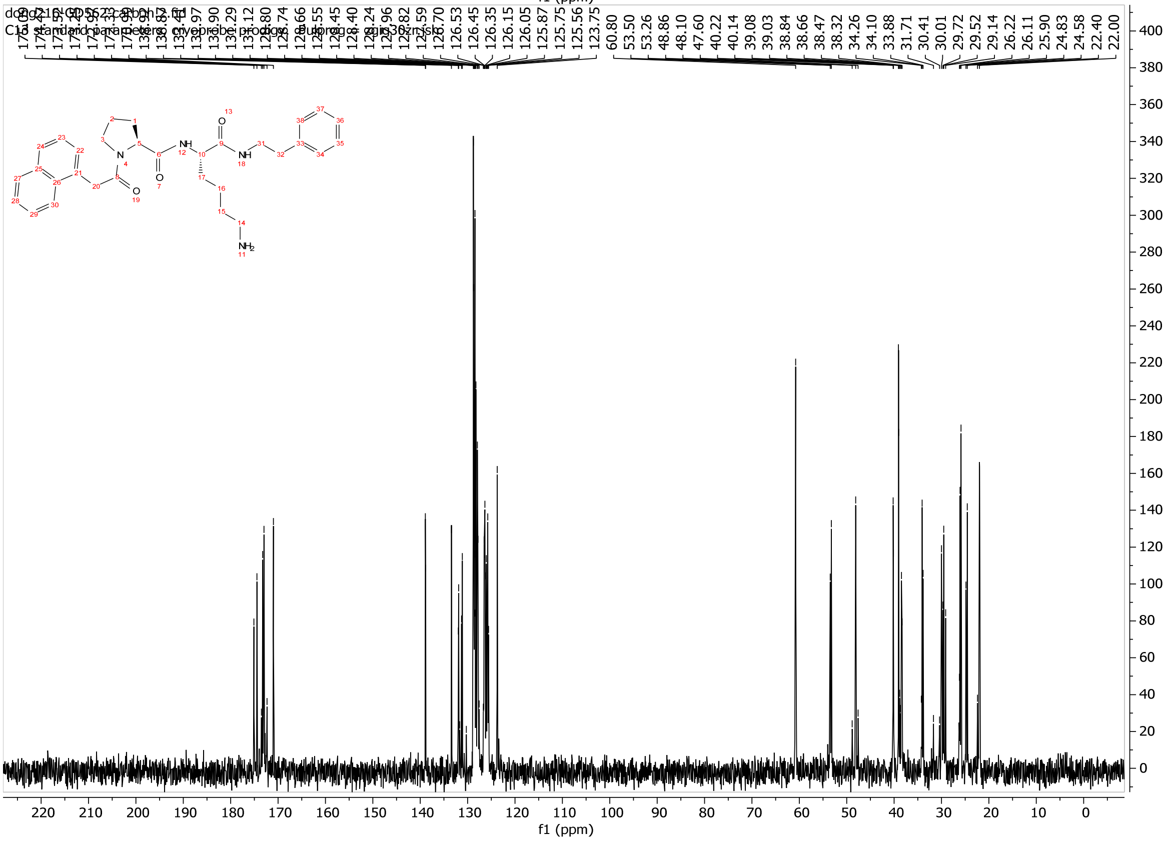
60.80
53.50
53.26
48.86
48.10
47.60
40.22
40.14
39.08
39.03
38.84
38.66
38.47
38.32
34.26
34.10
33.88
31.71
30.41
30.01
29.72
29.52
29.14
26.22
26.11
25.90
24.83
24.58
22.40
22.00
f1 (ppm)

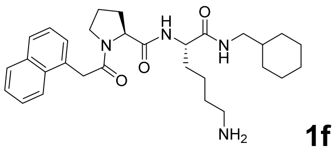

1f

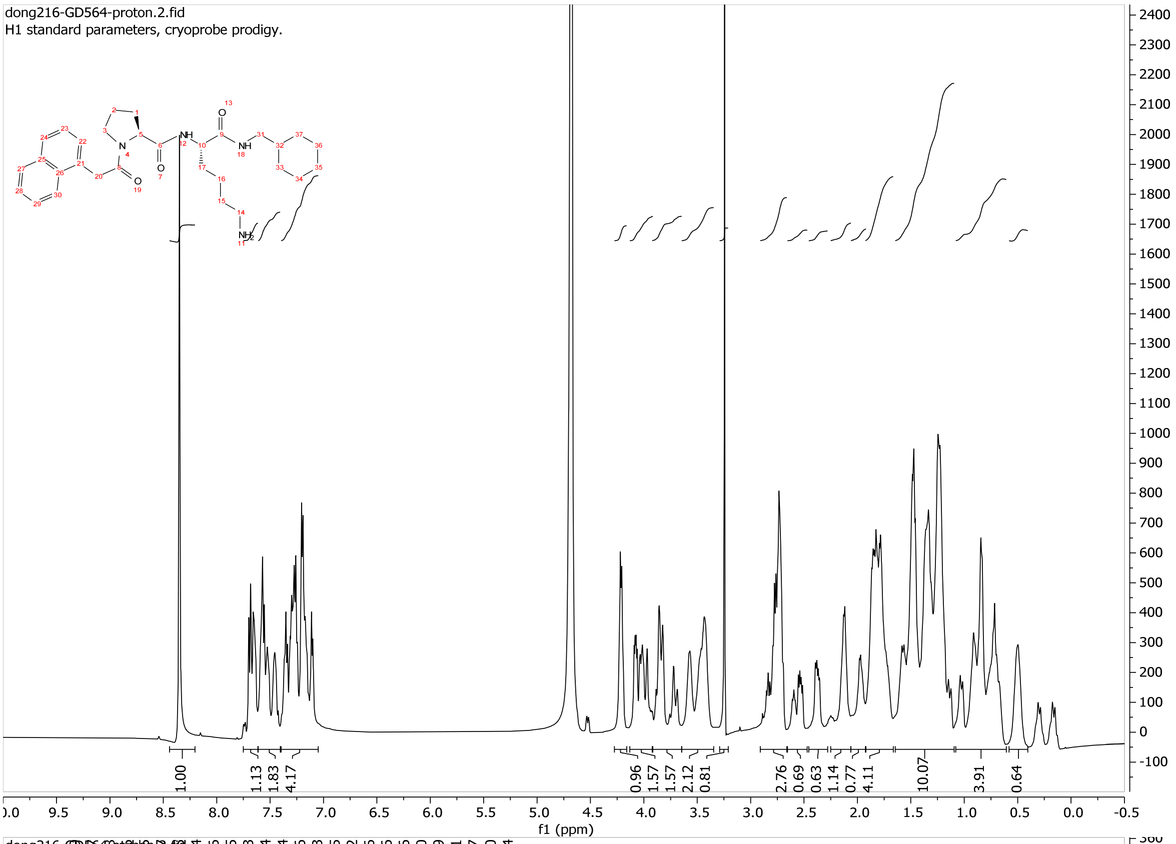
dong216-GD564-proton.2.fid
H1 standard parameters, cryoprobe prodigy.
1.00
1.13
1.83
4.17
0.96
1.57
1.57
2.12
0.81
2.76
0.69
0.63
1.14
0.77
4.11
10.07
3.91
0.64
f1 (ppm)

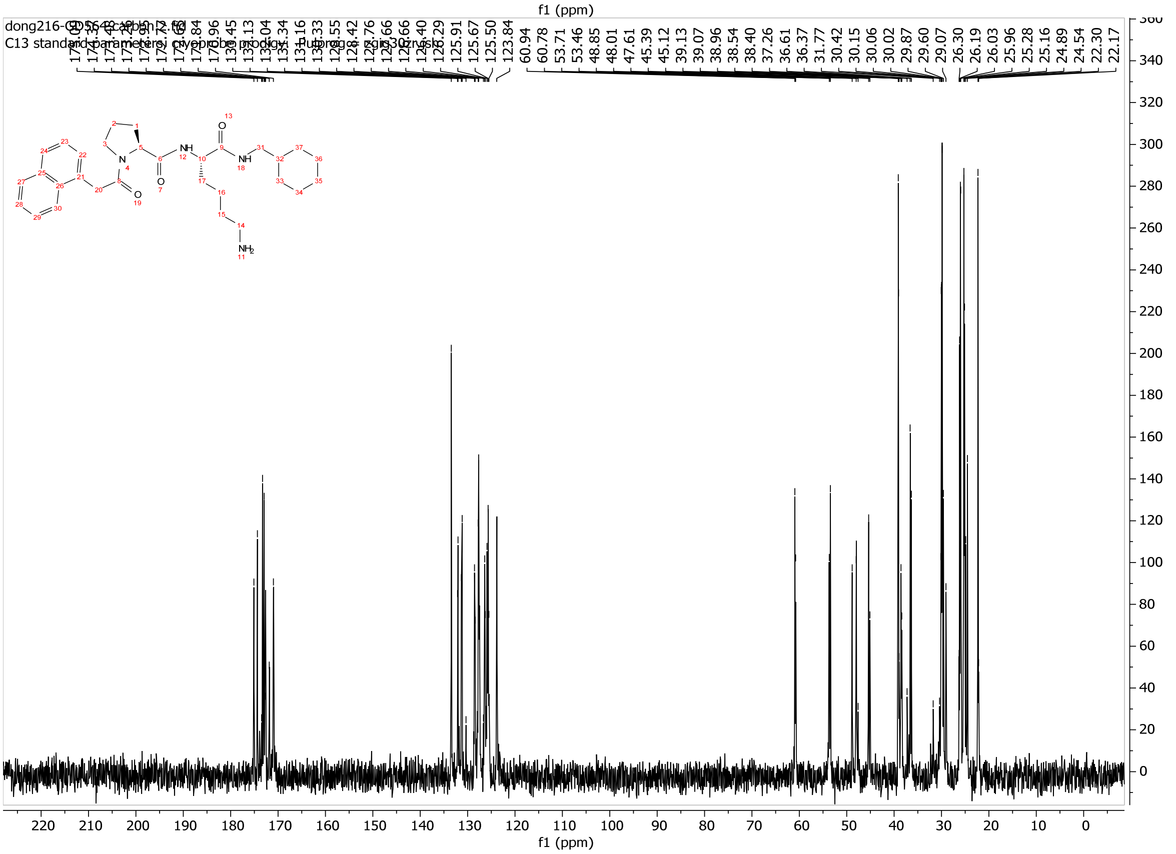
125.91
125.67
125.50
123.84
60.94
60.78
53.71
53.46
48.85
48.01
47.61
45.39
45.12
39.13
39.07
38.96
38.54
38.40
37.26
36.61
36.37
31.77
30.42
30.15
30.06
30.02
29.87
29.60
29.07
26.30
26.19
26.03
25.96
25.28
25.16
24.89
24.54
22.30
22.17
f1 (ppm)

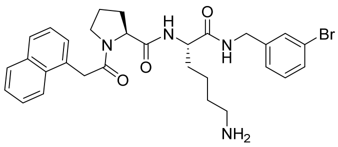

1g

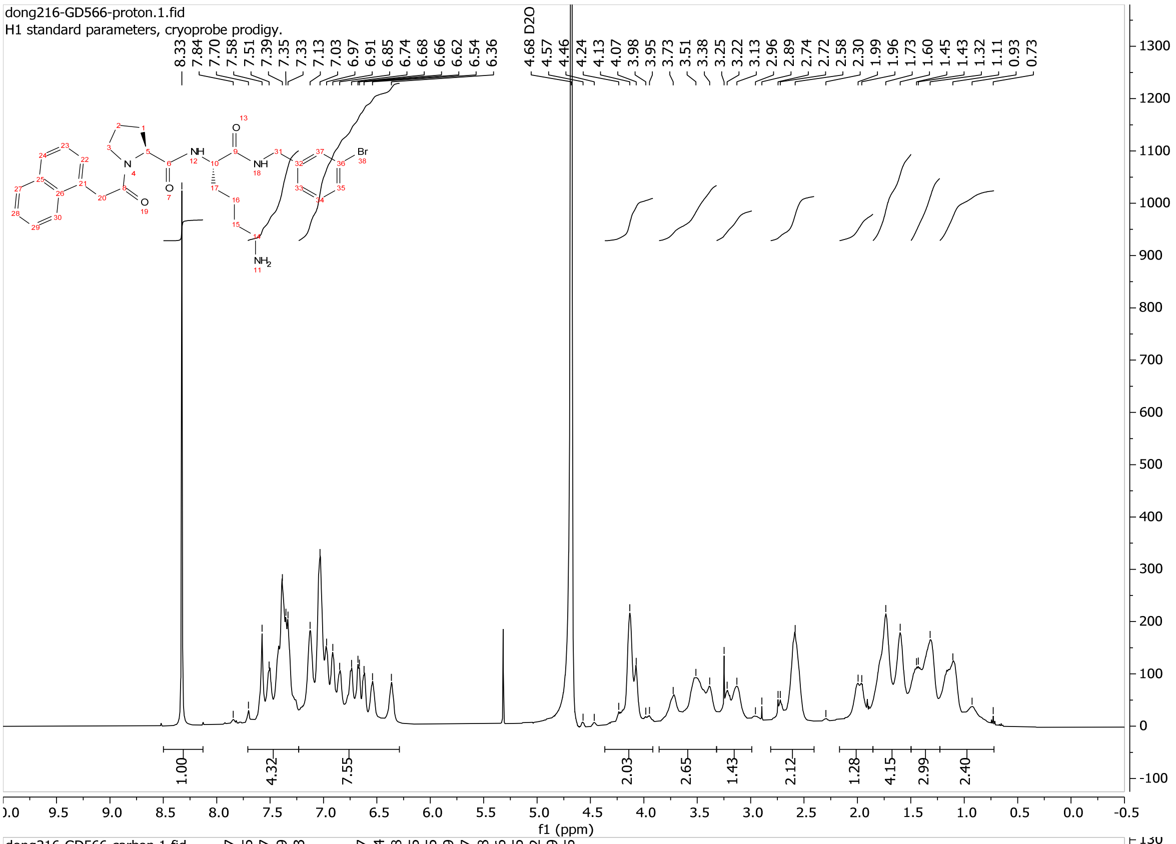
dong216-GD566-proton.1.fid
H1 standard parameters, cryoprobe prodigy.
8.33 7.84 7.70 7.58 7.51 7.39 7.35 7.33 7.13 7.03 6.97 6.91 6.85 6.74 6.68 6.66 6.62 6.54 6.36
4.68 D2O 4.57 4.46 4.24 4.13 4.07 3.98 3.95 3.73 3.51 3.38 3.25 3.22 3.13 2.96 2.89 2.74 2.72 2.58 2.30 1.99 1.96 1.73 1.60 1.45 1.43 1.32 1.11 0.93 0.73
1.00 4.32 7.55 2.03 2.65 1.43 2.12 1.28 4.15 2.99 2.40
f1 (ppm)

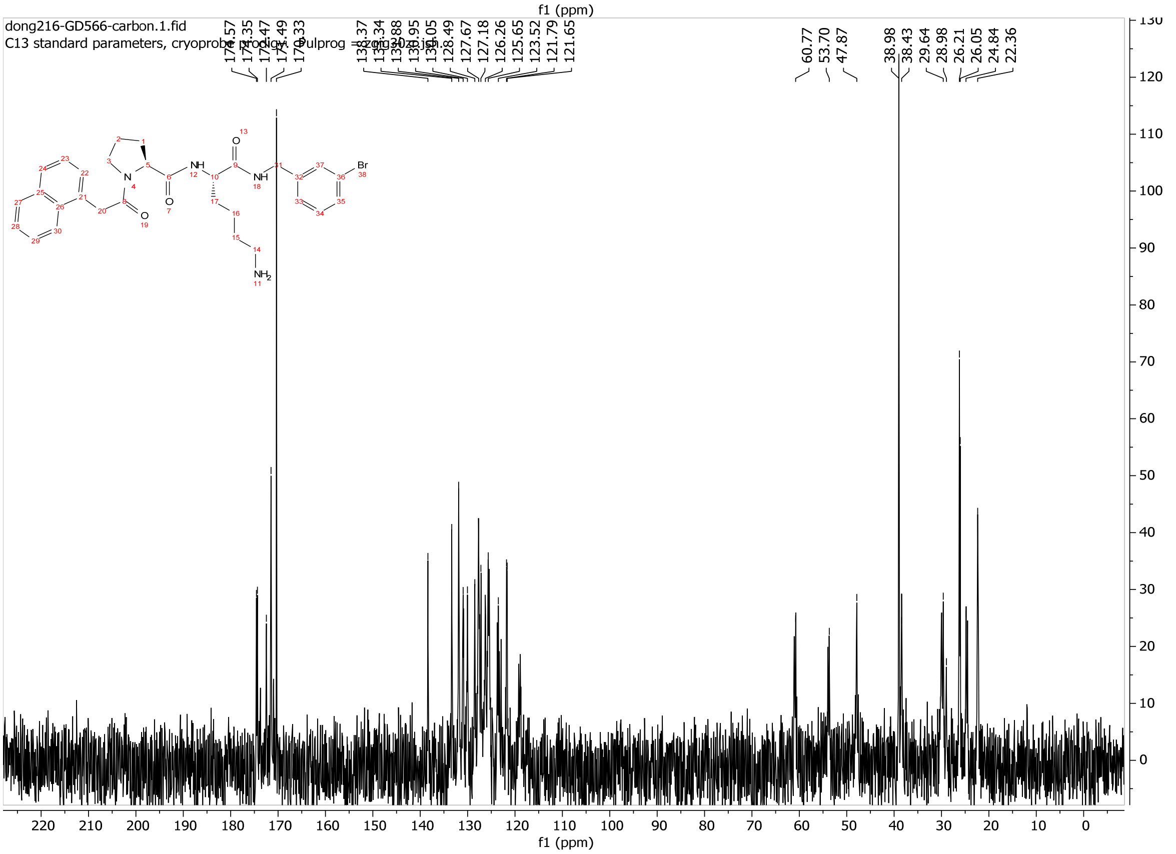
dong216-GD566-carbon.1.fid
C13 standard parameters, cryoprobe prodigy.
60.77 53.70 47.87 38.98 38.43 29.64 28.98 26.21 26.05 24.84 22.36
f1 (ppm)

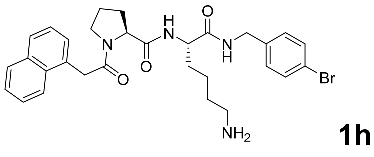

1h

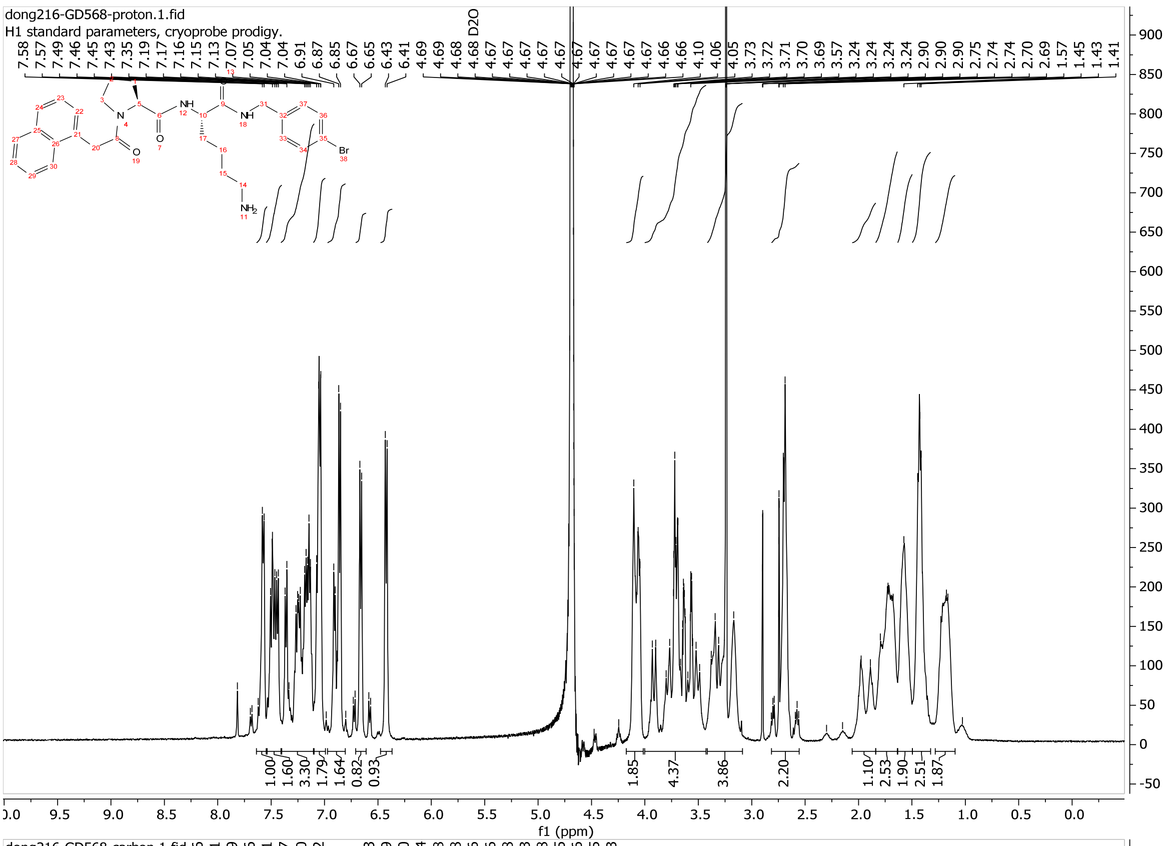
dong216-GD568-proton.1.fid
H1 standard parameters, cryoprobe prodigy.
D2O
f1 (ppm)

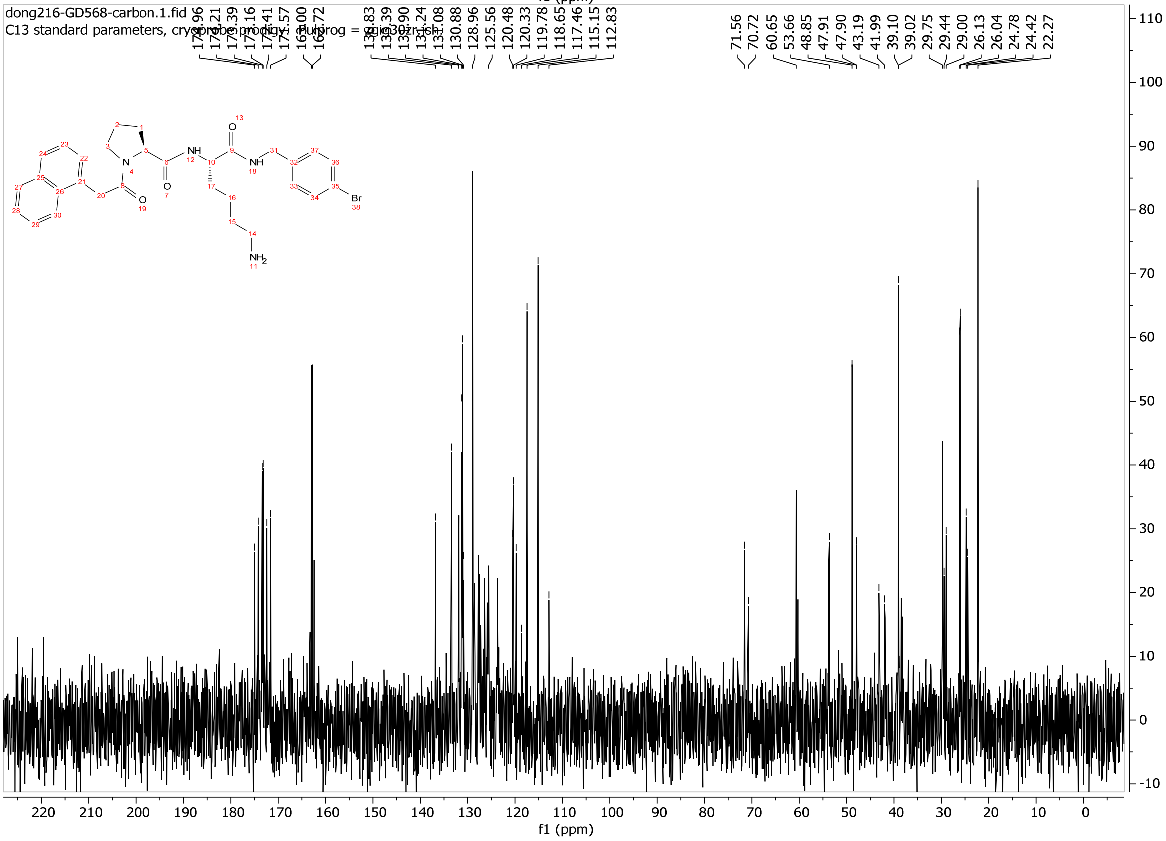
dong216-GD568-carbon.1.fid
C13 standard parameters, cryoprobe prodigy.
f1 (ppm)

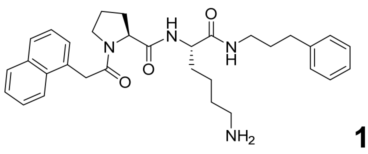
NH2

1e

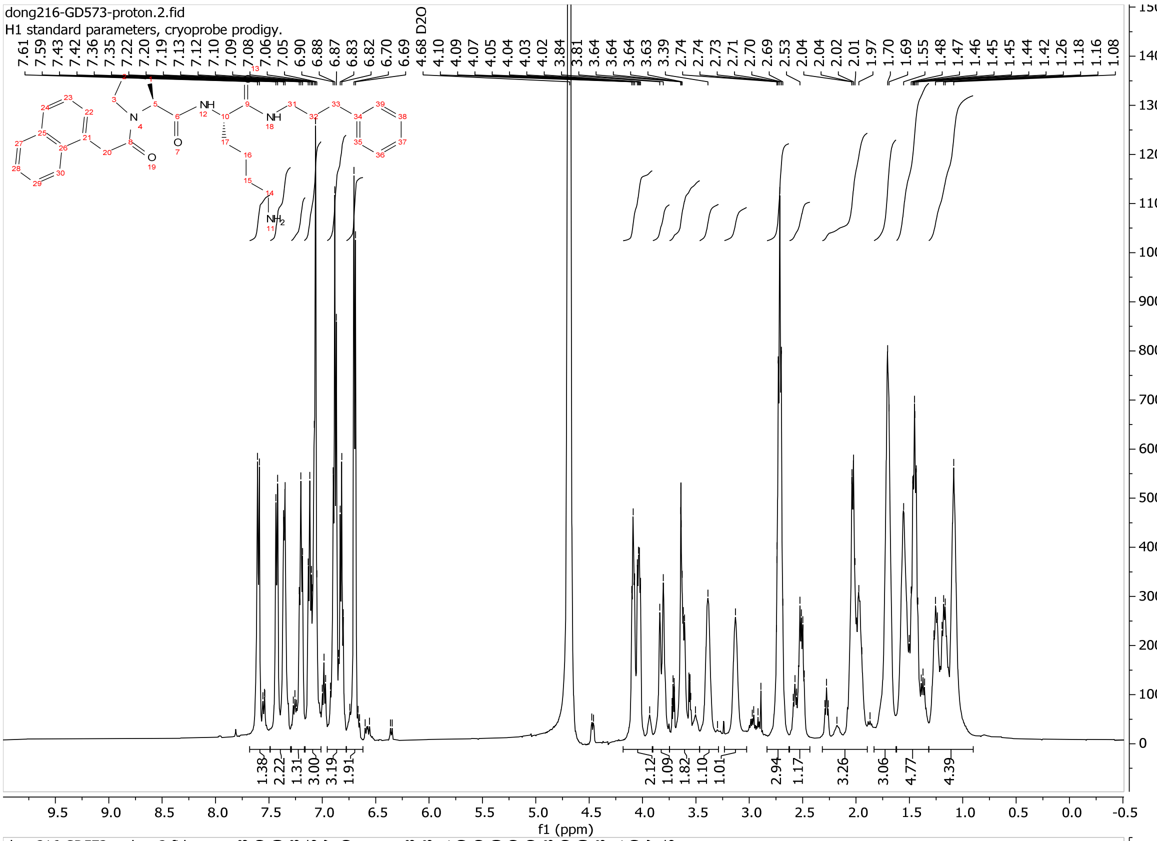
dong216-GD573-proton.2.fid
H1 standard parameters, cryoprobe prodigy.
f1 (ppm)

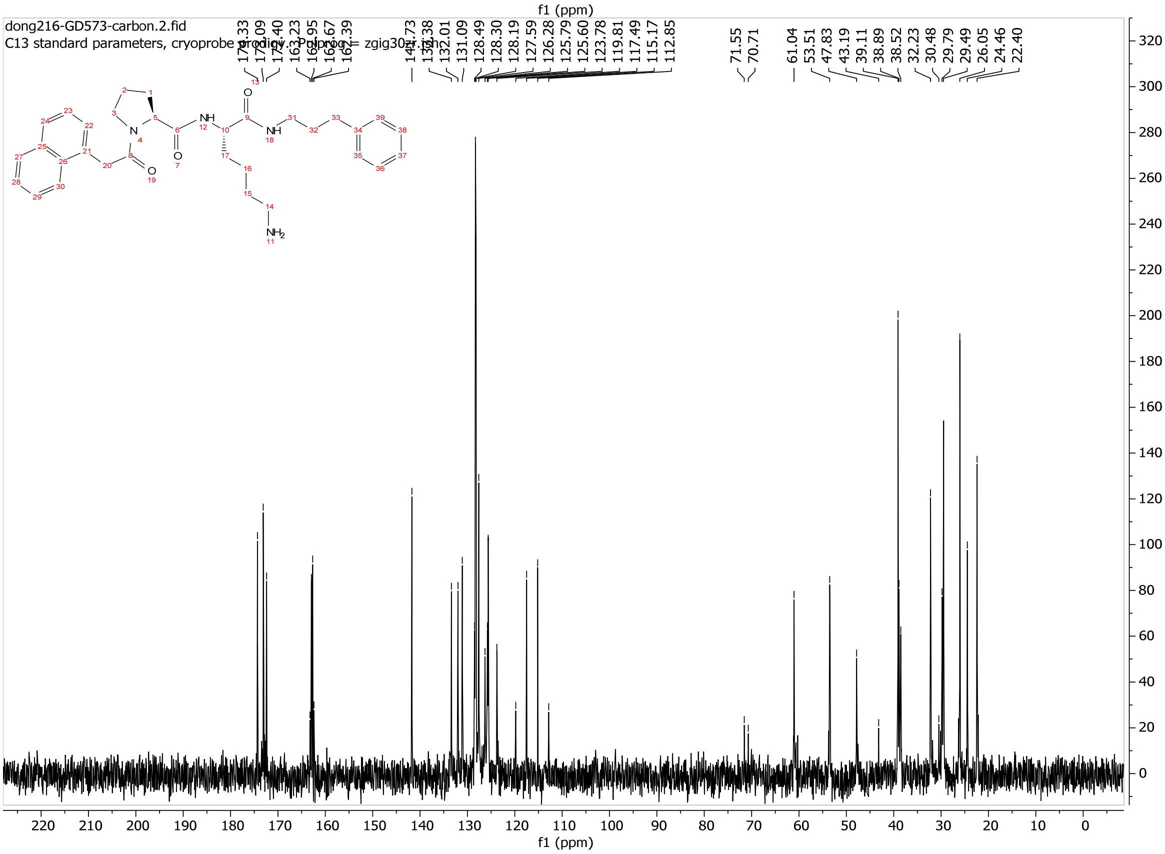
dong216-GD573-carbon.2.fid
C13 standard parameters, cryoprobe prodigy.
f1 (ppm)

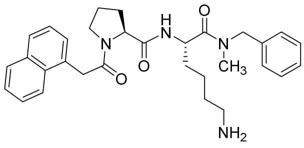

1i

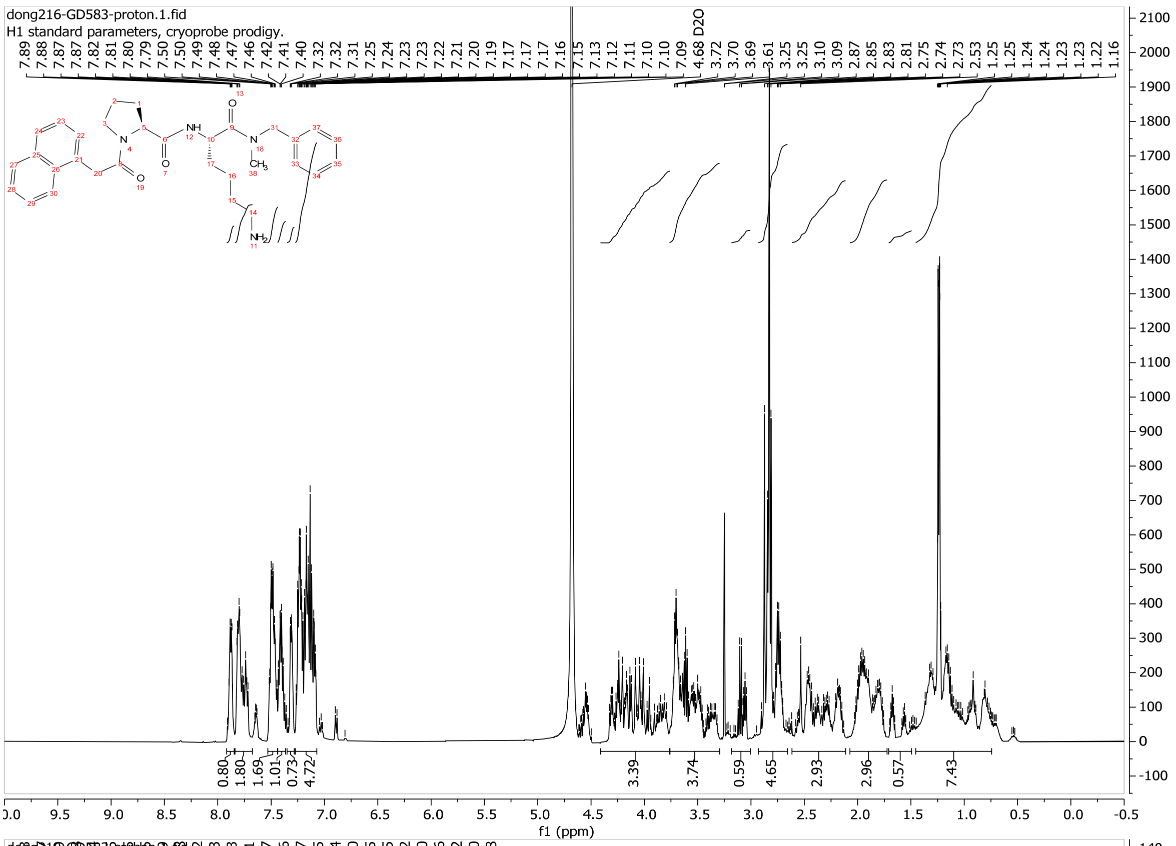
dong216-GD583-proton.1.fid
H1 standard parameters, cryoprobe prodigy.
f1 (ppm)

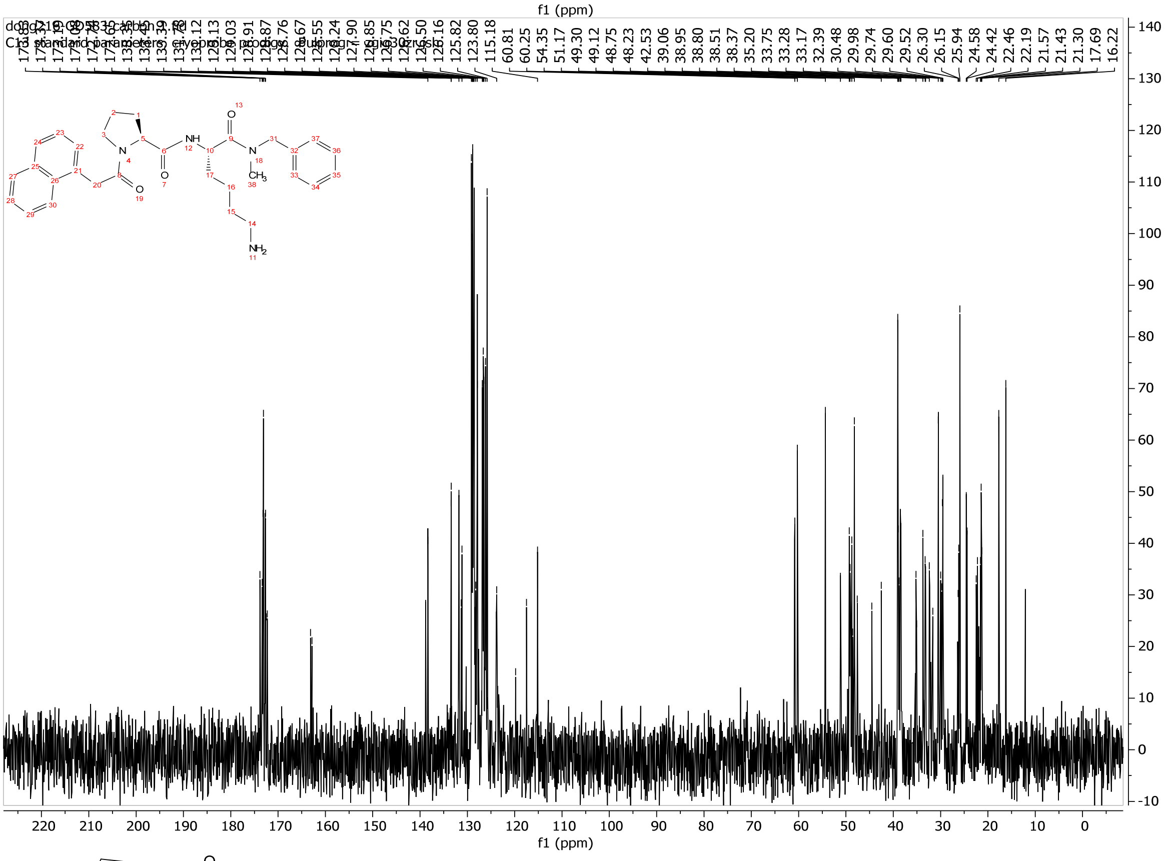
f1 (ppm)

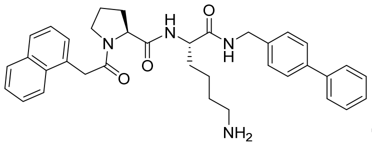

1j

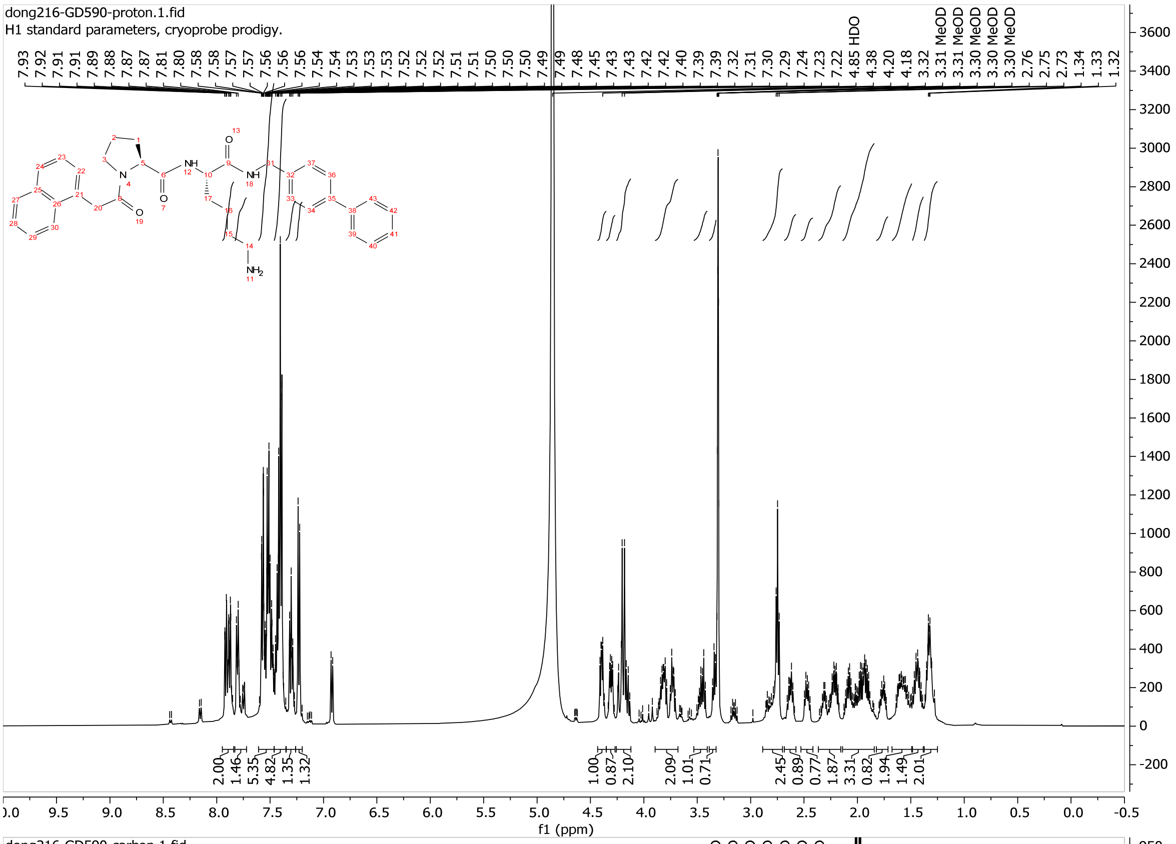
dong216-GD590-proton.1.fid
H1 standard parameters, cryoprobe prodigy.
7.93 7.92 7.91 7.91 7.89 7.88 7.87 7.87 7.81 7.80 7.58 7.58 7.57 7.57 7.56 7.56 7.56 7.54 7.54 7.53 7.53 7.53 7.52 7.52 7.52 7.51 7.51 7.50 7.50 7.49 7.49 7.48 7.45 7.43 7.43 7.42 7.42 7.40 7.39 7.39 7.32 7.31 7.30 7.29 7.24 7.23 7.22 4.85 HDO 4.38 4.20 4.18 3.32 3.31 MeOD 3.31 MeOD 3.30 MeOD 3.30 MeOD 3.30 MeOD 2.76 2.75 2.73 1.34 1.33 1.32
2.00 1.46 5.35 4.82 1.35 1.32 1.00 0.87 2.10 2.09 1.01 0.71 2.45 0.89 0.77 1.87 3.31 0.82 1.94 1.49 2.01
f1 (ppm)

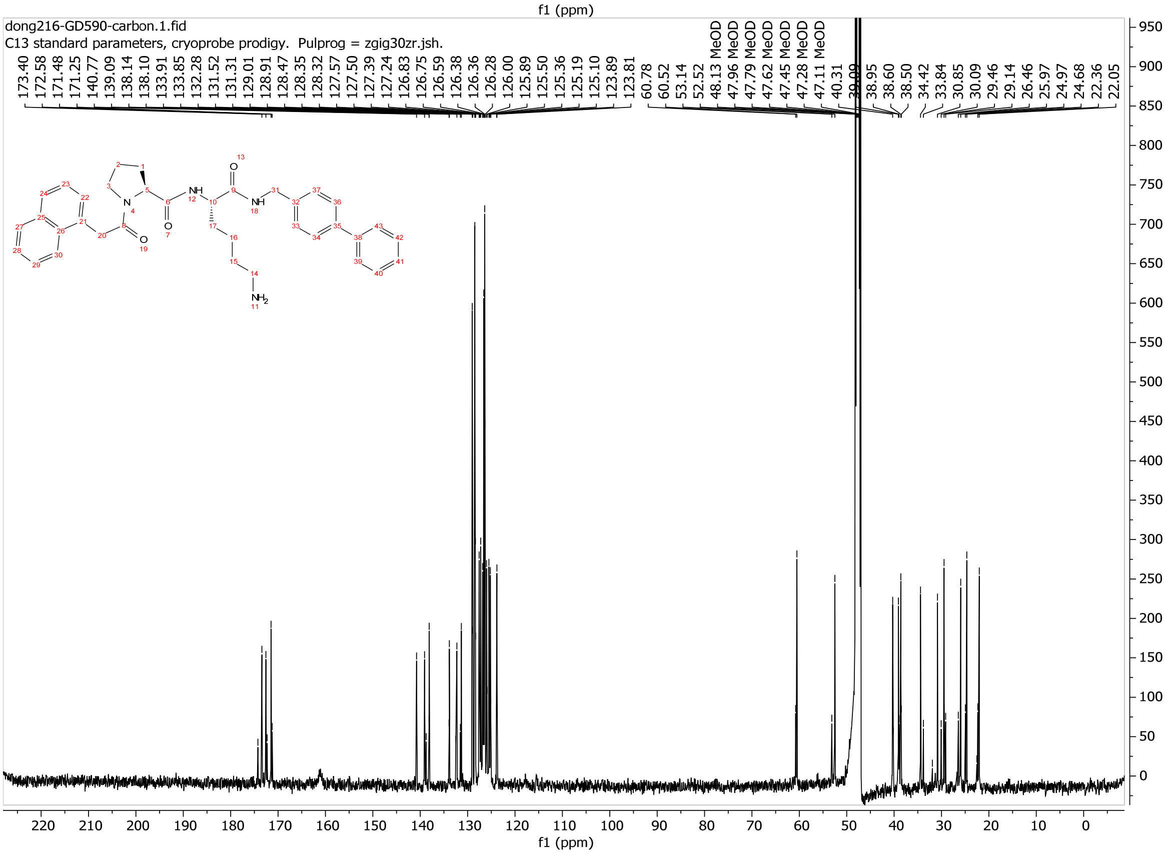
dong216-GD590-carbon.1.fid
C13 standard parameters, cryoprobe prodigy. Pulprog = zgig30zr.jsh.
173.40 172.58 171.48 171.25 140.77 139.09 138.14 138.10 133.91 133.85 132.28 131.52 131.31 129.01 128.91 128.47 128.35 128.32 127.57 127.50 127.39 127.24 126.83 126.75 126.59 126.38 126.36 126.28 126.00 125.89 125.50 125.36 125.19 125.10 123.89 123.81 60.78 60.52 53.14 52.52 48.13 MeOD 47.96 MeOD 47.79 MeOD 47.62 MeOD 47.45 MeOD 47.28 MeOD 47.11 MeOD 40.31 38.95 38.60 38.50 34.42 33.84 30.85 30.09 29.46 29.14 26.46 25.97 24.97 24.68 22.36 22.05
f1 (ppm)

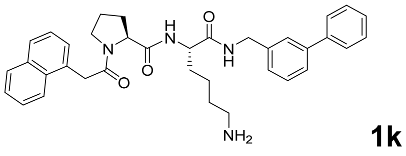

1k

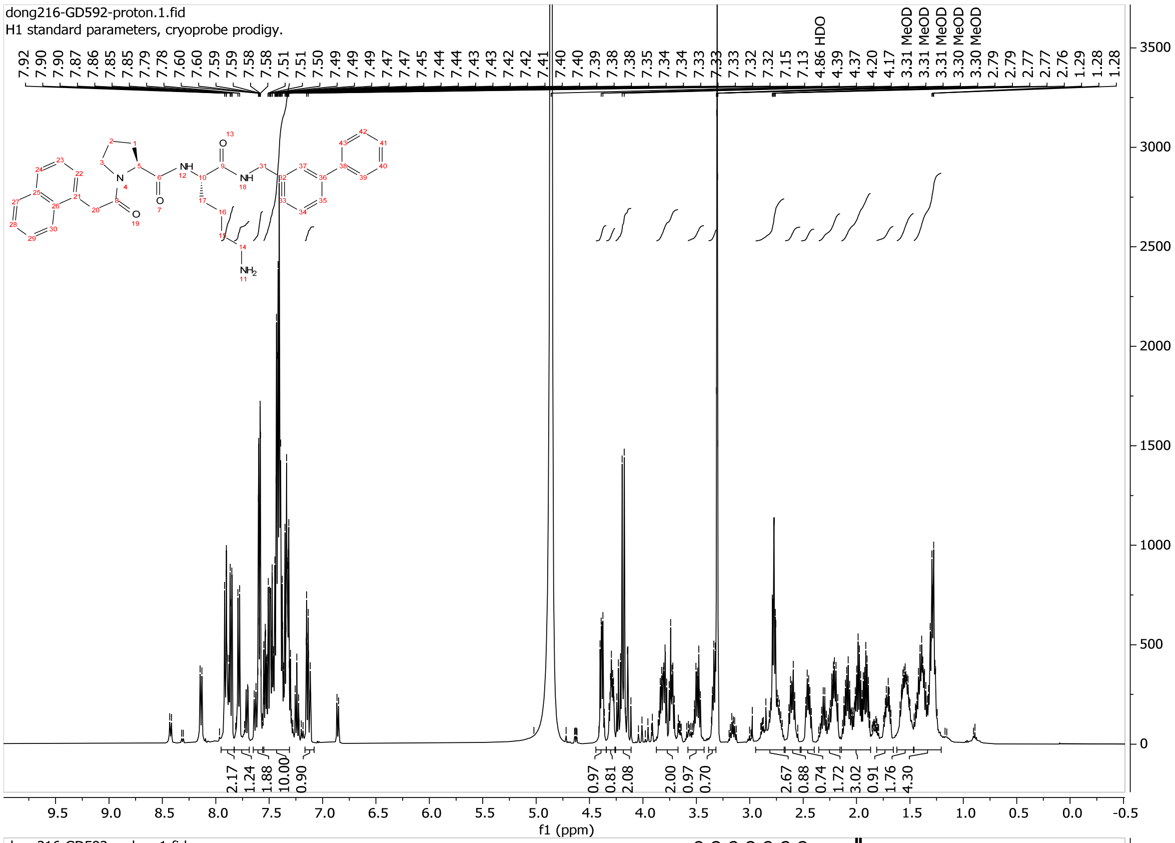
dong216-GD592-proton.1.fid
H1 standard parameters, cryoprobe prodigy.
7.92 7.90 7.90 7.87 7.86 7.85 7.85 7.79 7.78 7.60 7.59 7.59 7.58 7.58 7.51 7.51 7.50 7.49 7.49 7.49 7.47 7.47 7.45 7.44 7.44 7.43 7.43 7.42 7.42 7.41 7.40 7.40 7.39 7.38 7.38 7.35 7.34 7.34 7.33 7.33 7.33 7.32 7.32 7.15 7.13 4.86 HDO 4.39 4.37 4.20 4.17 3.31 MeOD 3.31 MeOD 3.31 MeOD 3.30 MeOD 3.30 MeOD 2.79 2.79 2.77 2.77 2.76 1.29 1.28 1.28
2.17 1.24 1.88 10.00 0.90 0.97 0.81 2.08 2.00 0.97 0.70 2.67 0.88 0.74 1.72 3.02 0.91 1.76 4.30
f1 (ppm)

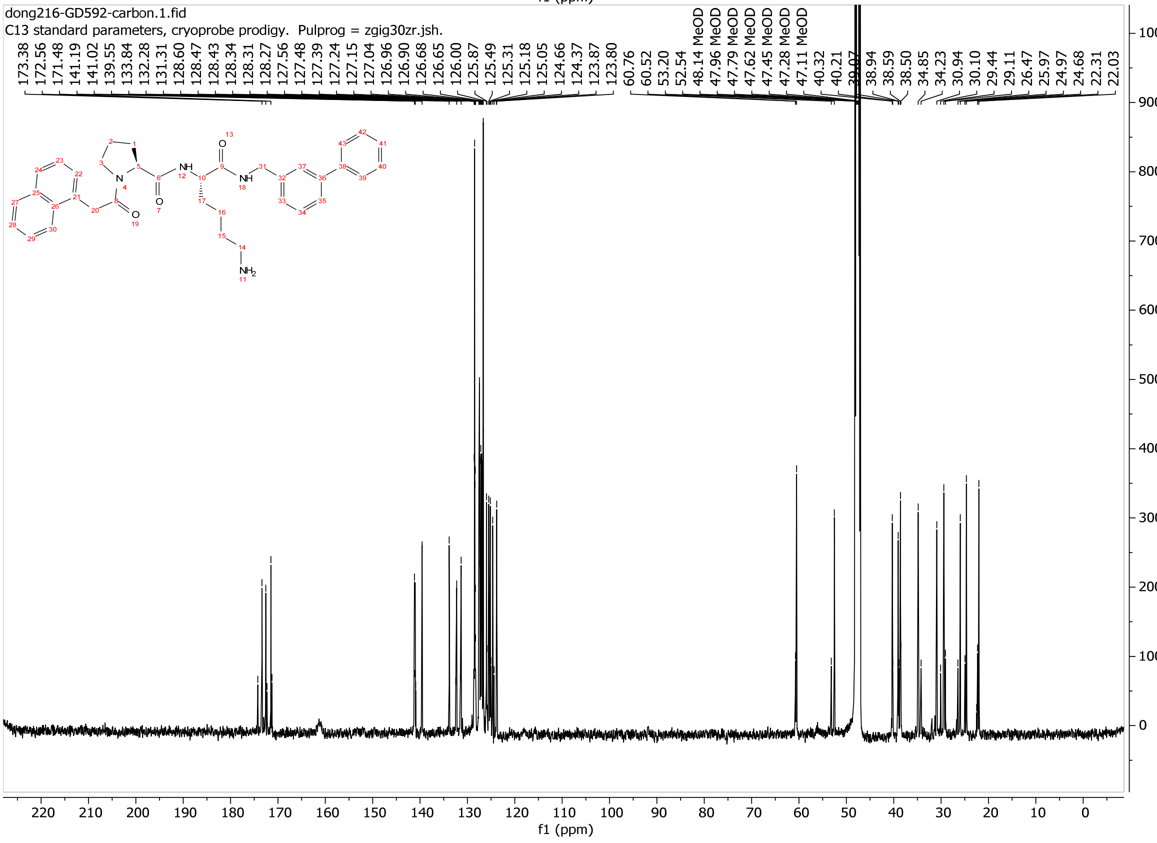
dong216-GD592-carbon.1.fid
C13 standard parameters, cryoprobe prodigy. Pulprog = zgig30zr.jsh.
173.38 172.56 171.48 141.19 141.02 139.55 133.84 132.28 131.31 128.60 128.47 128.43 128.34 128.31 128.27 127.56 127.48 127.39 127.24 127.15 127.04 126.96 126.90 126.68 126.65 126.00 125.87 125.49 125.31 125.18 125.05 124.66 124.37 123.87 123.80 60.76 60.52 53.20 52.54 48.14 MeOD 47.96 MeOD 47.79 MeOD 47.62 MeOD 47.45 MeOD 47.28 MeOD 47.11 MeOD 40.32 40.21 39.07 38.94 38.59 38.50 34.85 34.23 30.94 30.10 29.44 29.11 26.47 25.97 24.97 24.68 22.31 22.03
f1 (ppm)

Supplemental Table S1. MSλD Computed vs. Experimental Binding Free Energies (kcal/mol).

Ligand Name	Expt. $\Delta G_{bind} \pm \sigma$	MSλD $\Delta G_{bind} \pm \sigma$
**DC541**	-8.82 ± 0.07	-8.36 ± 0.24
**1a (GD556)**	-9.24 ± 0.04	-9.50 ± 0.24[a]
**1b (GD558)**	-9.20 ± 0.03	-8.71 ± 0.12[b]
**1c (GD560)**	-8.24 ± 0.03	-8.39 ± 0.18
**1d (GD562)**	-8.23 ± 0.04	-8.11 ± 0.18
**1e (GD573)**	-8.44 ± 0.01	-8.81 ± 0.22
**1f (GD564)**	-8.22 ± 0.03	-8.02 ± 0.13
**1g (GD566)**	-8.51 ± 0.04	-8.83 ± 0.33
**1h (GD568)**	-8.30 ± 0.07	-8.47 ± 0.14
**Mean Unsigned Error**		**0.28**
**Pearson R**		**0.71**

[a] with σ-hole lone pair ΔG_{bind}(**1b**) = -9.61 ± 0.40
[b] with σ-hole lone pair ΔG_{bind}(**1c**) = -8.70 ± 0.12

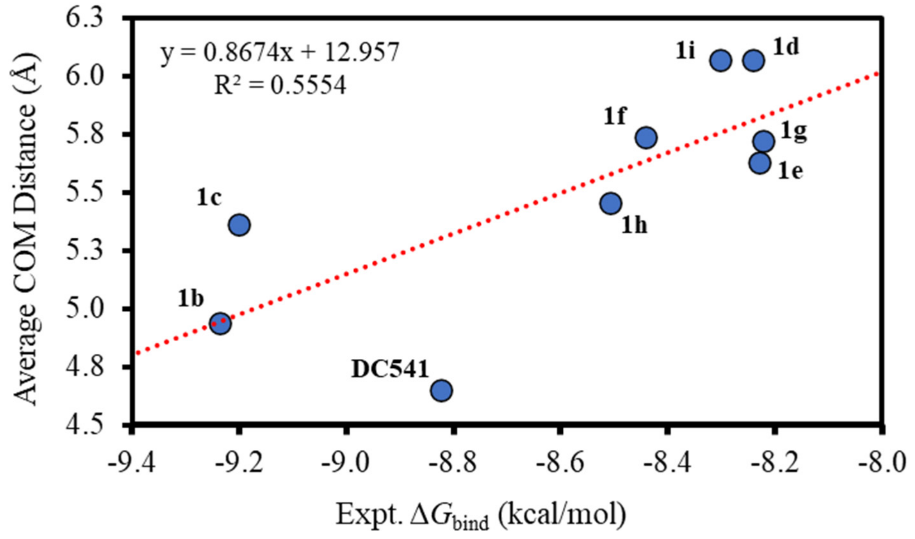

Supplemental Figure S1. Correlation between experimental binding affinities and average computed center of mass (COM) distances between C-region substituent rings and the phenyl ring of Tyr215.

References

1. Richardson, S. L.; Mao, Y.; Zhang, G.; Hanjra, P.; Peterson, D. L.; Huang, R. Kinetic mechanism of protein N-terminal methyltransferase 1. *J. Biol. Chem.* **2015,** 290, 11601-11610.
2. Dong, C.; Mao, Y.; Tempel, W.; Qin, S.; Li, L.; Loppnau, P.; Huang, R.; Min, J. Structural basis for substrate recognition by the human N-terminal methyltransferase 1. *Genes Dev.* **2015,** 29, 2343-2348.
3. Wu, H.; Min, J.; Lunin, V. V.; Antoshenko, T.; Dombrovski, L.; Zeng, H.; Allali-Hassani, A.; Campagna-Slater, V.; Vedadi, M.; Arrowsmith, C. H.; Plotnikov, A. N.; Schapira, M. Structural biology of human H3K9 methyltransferases. *PLoS One* **2010,** 5, e8570.
4. Feng, Y.; Xie, N.; Jin, M.; Stahley, M. R.; Stivers, J. T.; Zheng, Y. G. A transient kinetic analysis of PRMT1 catalysis. *Biochemistry* **2011,** 50, 7033-44.
5. Barsyte-Lovejoy, D.; Li, F.; Oudhoff, M. J.; Tatlock, J. H.; Dong, A.; Zeng, H.; Wu, H.; Freeman, S. A.; Schapira, M.; Senisterra, G. A.; Kuznetsova, E.; Marcellus, R.; Allali-Hassani, A.; Kennedy, S.; Lambert, J. P.; Couzens, A. L.; Aman, A.; Gingras, A. C.; Al-Awar, R.; Fish, P. V.; Gerstenberger, B. S.; Roberts, L.; Benn, C. L.; Grimley, R. L.; Braam, M. J.; Rossi, F. M.; Sudol, M.; Brown, P. J.; Bunnage, M. E.; Owen, D. R.; Zaph, C.; Vedadi, M.; Arrowsmith, C. H. (R)-PFI-2 is a potent and selective inhibitor of SETD7 methyltransferase activity in cells. *Proc Natl Acad Sci U S A* **2014,** 111, 12853-8.
6. Mackie, B. D.; Chen, D.; Dong, G.; Dong, C.; Parker, H.; Schaner Tooley, C. E.; Noinaj, N.; Min, J.; Huang, R. Selective Peptidomimetic Inhibitors of NTMT1/2: Rational Design, Synthesis, Characterization, and Crystallographic Studies. *J Med Chem* **2020,** 63, 9512-9522.
7. Debler, E. W.; Jain, K.; Warmack, R. A.; Feng, Y.; Clarke, S. G.; Blobel, G.; Stavropoulos, P. A glutamate/aspartate switch controls product specificity in a protein arginine methyltransferase. *Proc Natl Acad Sci U S A* **2016,** 113, 2068-73.
8. Chen, D.; Dong, G.; Noinaj, N.; Huang, R. Discovery of bisubstrate inhibitors for protein N-terminal methyltransferase 1. *J. Med. Chem.* **2019,** 62, 3773-3779.
9. Chen, D.; Dong, G.; Deng, Y.; Noinaj, N.; Huang, R. Structure-based Discovery of Cell-Potent Peptidomimetic Inhibitors for Protein N-Terminal Methyltransferase 1. *ACS Med Chem Lett* **2021,** 12, 485-493.
10. Kong, X.; Brooks, C. L. III λ Dynamics: A New Approach to Free Energy Calculations. *J. Chem. Phys.* **1996**, *105*, 2414-2423.
11. Knight, J. L.; Brooks, C. L. III Multisite λ Dynamics for Simulated Structure-Activity Relationship Studies. *J. Chem. Theory Comput.* **2011**, *7*, 2728-2739.
12. Pettersen, E. F.; Goddard, T. D.; Huang, C. C.; Couch, G. S.; Greenblatt, D. M.; Meng, E. C.; Ferrin, T. E. UCSF Chimera--a visualization system for exploratory research and analysis. *J. Comput. Chem.* **2004**, *25*, 1605-1612.
13. Best, R. B.; Mittal, J.; Feig, M.; MacKerell, A. D. Jr. Inclusion of Many-Body Effects in the Additive CHARMM Protein CMAP Potential Results in Enhanced Cooperativity of α-Helix And β-Hairpin Formation. *Biophys. J.* **2012**, *103*, 1045-1051.
14. Best, R. B.; Zhu, X.; Shim, J.; Lopes, P. E. M.; Mittal, J.; Feig, M.; MacKerell, A. D. Jr. Optimization of the Additive CHARMM All-Atom Protein Force Field Targeting Improved Sampling of the Backbone ϕ, ψ, and Side-Chain χ1 and χ2 Dihedral Angles. *J. Chem. Theory Comput.* **2012**, *8*, 3257-3273.
15. Vanommeslaeghe, K.; Hatcher, E.; Acharya, C.; Kundu, S.; Zhong, S.; Shim, J.; Darian, E.; Guvench, O.; Lopes, P.; Vorobyov, I.; MacKerell, A. D. Jr. CHARMM General Force Field: A Force Field for Drug-Like Molecules Compatible with the CHARMM All-Atom Additive Biological Force Fields. *J. Comput. Chem.* **2010**, *31*, 671-690.
16. Vanommeslaeghe, K.; MacKerell, A. D. Jr. Automation of the CHARMM General Force Field (CGenFF) I: Bond Perception and Atom Typing. *J. Chem. Inf. Model.* **2012**, *52*,

3144-3154.
17. Vanommeslaeghe, K.; Raman, E. P.; MacKerell, A. D. Jr. Automation of the CHARMM General Force Field (CGenFF) II: Assignment of Bonded Parameters and Partial Atomic Charges. *J. Chem. Inf. Model.* **2012**, *52*, 3155-3168.
18. Jorgensen, W. L.; Chandrasekhar, J.; Madura, J. D.; Impey, R. W.; Klein, M. L. Comparison of Simple Potential Functions for Simulating Liquid Water. *J. Chem. Phys.* **1983**, *79*, 926-935.
19. Jo, S.; Kim, T.; Iyer, V. G.; Im W. *J. Comput. Chem.* **2008**, *29*, 1859-1865.
20. Steinbach, P. J.; Brooks, B. R. New Spherical-Cutoff Methods for Long-Range Forces in Macromolecular Simulation. *J. Comp. Chem.* **1994**, *15*, 667-683.
21. Hayes, R. L.; Armacost, K. A.; Vilseck, J. Z.; Brooks, C. L. III Adaptive Landscape Flattening Accelerates Sampling of Alchemical Space in Multisite λ Dynamics. *J. Phys. Chem. B* **2017**, *121*, 3626-3635.
22. Keränen, H.; Pérez-Benito, L.; Ciordia, M.; Delgado, F.; Steinbrecher, T. B.; Oehlrich, D.; van Vlijmen, H. W. T.; Trabanco, A. A.; Tresadern, G. Acylguanidine Beta Secretase 1 Inhibitors: A Combined Experimental and Free Energy Perturbation Study. *J. Chem. Theory Comput.* **2017**, *13*, 1439-1453.
23. The PyMOL Molecular Graphics System, Version 1.8, Schrodinger LLC, New York, New York, United States.
24. Kansy, M.; Senner, F.; Gubernator, K. Parallel Artificial Membrane Permeation Assay in the Description of Passive Absorption Processes. *J. Med. Chem.* **1998**, *41* (7), 1007–1010.
25. Avdeef, A.; Strafford, M.; Block, E.; Balogh, M. P.; Chambliss, W.; Khan, I. Drug Absorption in Vitro Model: Filter-Immobilized Artificial Membranes: 2. Studies of the Permeability Properties of Lactones in Piper Methysticum Forst. *Eur. J. Pharm. Sci.* **2001**, *14* (4), 271–280. https://doi.org/10.1016/S0928-0987(01)00191-9.
